# Supplementary material for: Exploring Applications of Artificial Intelligence Tools in Clinical Care and Health Professions Education: An Online Module for Students
Source: MedEdPORTAL. 2025 May 1;21:11524. doi: 10.15766/mep_2374-8265.11524 (PMC12043951; doi:10.15766/mep_2374-8265.11524)
Supplement: Supplementary file 1 — AI in Medicine folderPre- and Posttest.docxFeedback Survey.docx [file mep_2374-8265.11524-s001.zip › A. AI in Medicine/assets/Reflective Questions (Interactive PDF Form).pdf]

Reflective Questions

Instructions: *Please write a reflection based on the following questions:*

1. **How do you think AI can enhance the care of a patient in the future?**

2. **What do you think are your innately human skills that will not be replicated by AI?**

3. **How do you think AI will change the dynamic of interprofessional healthcare teams?**

4. **How will you remain current with the evolving landscape of artificial intelligence?**
