# Supplementary material for: Exploring Applications of Artificial Intelligence Tools in Clinical Care and Health Professions Education: An Online Module for Students
Source: MedEdPORTAL. 2025 May 1;21:11524. doi: 10.15766/mep_2374-8265.11524 (PMC12043951; doi:10.15766/mep_2374-8265.11524)
Supplement: Supplementary file 1 — AI in Medicine folderPre- and Posttest.docxFeedback Survey.docx [file mep_2374-8265.11524-s001.zip › A. AI in Medicine/index.html]

Applications of Artificial Intelligence (AI) Tools in Healthcare
