# Supplementary material for: Exploring Applications of Artificial Intelligence Tools in Clinical Care and Health Professions Education: An Online Module for Students
Source: MedEdPORTAL. 2025 May 1;21:11524. doi: 10.15766/mep_2374-8265.11524 (PMC12043951; doi:10.15766/mep_2374-8265.11524)
Supplement: Supplementary file 1 — AI in Medicine folderPre- and Posttest.docxFeedback Survey.docx [file mep_2374-8265.11524-s001.zip › B. Pre- and Posttest.docx]

# Appendix B: Pre-Test/Post-Test Questions (These questions and rationales are already embedded within the module. An editable version is provided here for educators for easy access and review. Time for pre-test and post-test should be less than 10 minutes.)

1. What is Machine Learning (ML)?
   1. A type of artificial intelligence that improves with experience
   2. The process of programming computers to follow specific rules
   3. A subfield of computer science focused on the development of intelligent machines
   4. A technology used solely for data analysis

**Rationale:** Machine Learning (ML) is a subset of artificial intelligence (AI) where algorithms learn from data to make predictions or decisions without being explicitly programmed for every scenario. Unlike traditional programming that follows specific rules (incorrect option), ML systems adapt and improve as they process more data. It’s not solely a technology for data analysis or the broader field of computer science (incorrect options), but a process that specifically enables computer systems to learn over time.

1. Which of the following is an example of AI not using machine learning?
   1. Google’s early search algorithm
   2. AlphaGo defeating a professional Go player
   3. IDx-DR detecting diabetic retinopathy
   4. AI applications in self-driving cars

**Rationale:** Google’s early search algorithms relied on predefined rules and keyword matching rather than machine learning (ML). AlphaGo, IDx-DR, and self-driving car applications all use ML algorithms to recognize patterns, make decisions, and improve based on data. Early search algorithms didn’t have this adaptability, distinguishing them as non-ML AI.

1. What is Generative AI (GenAI) primarily used for?
   1. Enhancing human intelligence
   2. Synthesizing new content like text, images, videos, and audio
   3. Creating virtual reality environments
   4. Solving complex mathematical problems

**Rationale:** Generative AI (GenAI) models are designed to generate new content, whether it be text, images, or other media types. GenAI does not directly create VR environments or solve complex mathematical problems (incorrect options); instead, it is particularly valued for its ability to synthesize content.

1. How does Artificial Intelligence (AI) enhance the experience in Virtual Reality (VR) environments?
   1. By allowing physical interaction with the virtual environment
   2. By creating intelligent characters and using natural language processing for interaction
   3. By enabling real-world object detection
   4. By projecting digital elements onto the real world

**Rationale:** In VR, AI is often used to create realistic, interactive elements such as intelligent characters that users can interact with naturally. Unlike physical interaction or object detection, which focus on sensing, AI’s primary function in VR environments is to enhance immersion and realism through intelligent and responsive interactions. Physical interaction in VR, like using hand controllers or VR gloves, is more of a hardware feature than an AI function. AI’s role in VR is typically about creating realistic, responsive virtual elements, rather than enabling direct physical interaction with the digital environment. Real-world object detection is more commonly associated with Augmented Reality (AR), where digital information is layered onto the real world based on the objects around the user. In VR, the environment is entirely digital, so there’s less emphasis on detecting real-world objects, which are not present in the fully immersive virtual space. Projecting digital elements onto the real world describes AR rather than VR. VR completely replaces the real-world surroundings with a digital environment, so digital elements are confined to the virtual space and are not projected onto the real world.

1. In Augmented Reality (AR), what role does AI play?
   1. It creates a fully immersive environment separate from the real world.
   2. It is used for creating virtual reality headsets.
   3. It assists in image recognition and object detection to overlay digital information accurately.
   4. AI is not used in AR.

**Rationale:** AI in AR typically supports the recognition of physical objects and their spatial positioning, allowing digital overlays to be accurately displayed. Unlike creating a fully immersive environment or VR headsets (incorrect options), AI’s role in AR centers on processing real-world visuals to display relevant digital information in real-time.

1. What is a key feature of Mixed Reality (MR) that differentiates it from AR and VR?
   1. It is a fully digital environment with no real-world elements.
   2. It involves the co-existence and interaction of physical and digital objects in real-time.
   3. It only overlays digital information without interaction.
   4. It uses AI solely for voice recognition.

**Rationale:** Mixed Reality (MR) uniquely combines elements of the real and digital worlds, enabling users to interact with digital objects as if they were part of the physical environment. This differentiates MR from AR (which merely overlays information) and VR (which is fully digital without real-world interaction). Voice recognition, a more general AI function, isn’t a defining feature of MR.

1. Which of the following is an example of an application of AI in healthcare?
   1. Interpretation of imaging studies without any human input
   2. Replacement of human interaction in psychological counseling
   3. Identification of appropriate diagnosis codes for medical claims
   4. Automating the entire decision-making process in surgical procedure

**Rationale:** AI is commonly used to streamline administrative tasks, such as medical coding. Fully autonomous interpretation of imaging studies or decision-making in surgery is rare, as these applications generally involve human oversight. Psychological counseling also relies heavily on human interaction, making replacement by AI inappropriate.

1. What is a major application of AI in electronic health records (EHRs)?
   1. Eliminating the need for patient records
   2. Replacing doctors in decision making
   3. Voice-to-text translation for efficient documentation
   4. Predicting future health outcomes with 100% accuracy

**Rationale:** AI assists with the transcription of spoken information into text, improving the efficiency of documentation in EHRs. AI doesn’t eliminate the need for patient records or replace doctors (incorrect options) and cannot predict health outcomes with perfect accuracy, though it can assist with risk stratification.

1. A primary care clinic employs an AI tool for remote patient monitoring in chronic disease management. The main purpose of this AI tool is to:
   1. Automate medication prescription.
   2. Replace the need for in-person doctor visits.
   3. Assist in managing patient care outside the healthcare setting.
   4. Perform all functions of a primary care physician.

**Rationale:** AI tools for remote patient monitoring are designed to support the ongoing management of patients’ health outside the clinic. These tools help track health metrics (e.g., blood pressure, glucose levels) and provide data to inform clinical decisions, enhancing care without replacing necessary in-person visits or automating physician tasks. The tool doesn't replace physicians but instead supports patients in their daily lives, allowing proactive management of chronic conditions.

1. In a healthcare system, AI is used to streamline the reimbursement process. This AI application is primarily focused on:
   1. Replacing all administrative staff.
   2. Improving the efficiency and effectiveness of administrative processes.
   3. Diagnosing diseases for insurance purposes.
   4. Predicting future healthcare costs for patients.

**Rationale:** AI applications in reimbursement aim to automate and improve administrative workflows, such as claims processing, coding, and identifying potential billing errors. This reduces manual tasks and minimizes errors, streamlining the process rather than replacing all staff or predicting healthcare costs, which are separate applications. Diagnosing diseases for insurance purposes isn’t typically a function of AI in reimbursement, as diagnosis is a clinical rather than an administrative function.
