# Supplementary material for: Exploring Applications of Artificial Intelligence Tools in Clinical Care and Health Professions Education: An Online Module for Students
Source: MedEdPORTAL. 2025 May 1;21:11524. doi: 10.15766/mep_2374-8265.11524 (PMC12043951; doi:10.15766/mep_2374-8265.11524)
Supplement: Supplementary file 1 — AI in Medicine folderPre- and Posttest.docxFeedback Survey.docx [file mep_2374-8265.11524-s001.zip › C. Feedback Survey.docx]

**Appendix C: Artificial Intelligence in Healthcare Module Feedback Survey**

**This appendix is provided to educators if they wish to edit and deliver their own survey after completion of the module. The time to complete the survey should be less than 5 minutes.**

Dear Participant,

Thank you for completing the module. Your feedback is essential for us to improve the quality of our educational resources. Please take a few minutes to provide your honest opinions and suggestions.

1. Please rate the overall quality of the AI in Medicine module on a scale from 1 to 5, with 1 being poor and 5 being excellent. [Likert Scale 1-5 (1-Poor, 2- Fair, 3-Good, 4- Very Good, 5-Excellent]
2. What aspects of the module's content did you find most valuable or interesting? [OPEN TEXT BOX]
3. Were there any specific topics or concepts that you found confusing or difficult to understand? If so, please describe. [OPEN TEXT BOX]
4. Were the visuals, graphics, and multimedia elements used in the module effective in enhancing your understanding?  [YES/NO] (If NO, please let us know what would be helpful to enhance your understanding.) [OPEN TEXT BOX]
5. Do you have any other suggestions for improving the clarity and presentation of the module? [OPEN TEXT]
6. Did you feel this module has applicability to your future career? [OPEN TEXT BOX]
7. How much time did it take for you to complete this module? Less than 15 minutes, 15-30 minutes More than 30 minutes

Thank you for participating in this survey! Your feedback is valuable to us in improving our educational resources.
