# Supplementary material for: Exploring Applications of Artificial Intelligence Tools in Clinical Care and Health Professions Education: An Online Module for Students
Source: MedEdPORTAL. 2025 May 1;21:11524. doi: 10.15766/mep_2374-8265.11524 (PMC12043951; doi:10.15766/mep_2374-8265.11524)
Supplement: Supplementary file 1 — AI in Medicine folderPre- and Posttest.docxFeedback Survey.docx [file mep_2374-8265.11524-s001.zip › _Educational Summary Report_11524.pdf]

MedEdPORTAL® The AAMC Journal of Teaching and Learning Resources

Original Publication Open Access

Exploring Applications of Artificial Intelligence Tools in Clinical Care and Health Professions Education: An Online Module for Students

Gauri Agarwal, MD*, Lokesh Ramamoorthi, MS, MBA, Trevor Yuen, MS, Edwin Merced, EdD, Jacqueline Brenner, Winfred Wu, MD, MPH, Richard Sabina, PhD

*Corresponding author: gagarwal@med.miami.edu

Abstract

Introduction: Health professions schools vary widely in integration of artificial intelligence (AI) into their curriculum, and freely available instructional modules covering AI's applications and implications are lacking. Health professions students need to understand AI's impact on patient care, research, and education. **Methods:** We developed an interactive 30-minute asynchronous, self-paced online module for medical, nursing, and physical therapy students at our institution. The module, built on the Articulate 360 platform for easy dissemination to other schools, comprises videos, tests, and a list of resources for continued learning. It was initially implemented with 200 first-year medical students, who completed pre- and postmodule tests to assess knowledge gain and a feedback survey to assess the module. **Results:** A total of 164 students completed both the pre- and postmodule tests, and 144 completed the feedback survey. The mean percentage of students with correct test responses improved pre- to postmodule from 74% to 87% ($p < .001$), indicating significant knowledge gain. Feedback comments highlighted the module's relevance, manageable completion time, and video content. Suggestions for improvement included having more interactive elements and providing detailed explanations on complex concepts such as virtual reality and the ethics of AI. **Discussion:** The AI module successfully enhanced knowledge and was well received. Future iterations will incorporate more video content and improved sections on virtual reality and ethics. The module's adaptability and ease of integration make it a valuable resource for other health professions schools and allow students to be informed about the evolving field of AI in health care.

Keywords
Artificial Intelligence, Interprofessional Education, Virtual Reality, Machine Learning, Self-Directed/Online Learning

Educational Objectives

By the end of this activity, learners will be able to:

1. Define terms such as artificial intelligence (AI), machine learning, deep learning, and generative AI.
2. Describe the applications of AI to clinical health care.
3. Describe the applications of AI to health professions education and training.
4. Reflect on the ethical and humanistic implications of using AI tools.
5. Reflect on the future integration of AI tools.
6. Review case studies of the use of AI in clinical care and education.

Citation:
Agarwal G, Ramamoorthi L, Yuen T, et al. Exploring applications of artificial intelligence tools in clinical care and health professions education: an online module for students. *MedEdPORTAL*. 2025;21:11524. https://doi.org/10.15766/mep_2374-8265.11524

Introduction

Health professions schools vary widely in the depth and length of exposure to artificial intelligence (AI) within the curriculum. The AI field is growing rapidly, and health professions students need to understand its impact on the care of their patients.[1,2] There are existing concerns that health care team members may become over reliant on AI algorithms, leading to a loss of core competencies such as medical knowledge, communication skills, professionalism, and empathy. AI algorithms could provide biased or inaccurate recommendations and result in the misuse of confidential patient data.[3-6] Alternatively, AI could be used to improve communication and increase time at the bedside by managing mundane but important tasks, such as documenting encounters and providing reminders about appointments.[3-6] Automating these tasks could also

Copyright © 2025 Agarwal et al. This is an open-access publication distributed under the terms of the Creative Commons Attribution license.

improve the well-being of health care professionals, allowing them to provide more empathetic care.[2,6] AI could personalize care in a meaningful way, by assisting physicians and nurses in tailoring recommendations and education to the individual patient.[2,6]

A scoping review in 2021 by Lee and colleagues[7] identified a diverse range of 22 published studies on the use of AI in undergraduate medical education, but only a few studies offered specific curricular recommendations. The review discussed barriers to introducing AI within the curriculum, including faculty resistance, lack of faculty expertise, lack of AI accreditation and licensing requirements, and limited curricular hours.[7] In a few case studies of piloted programs of AI curriculum, reported outcomes included student satisfaction, knowledge acquisition, and skill transfer.[7] With the launch of ChatGPT in 2022, the number of medical education publications related to AI increased exponentially. However, a scoping review in 2024 found only four articles that emphasized foundational concepts in AI for medical students, and all four articles described content delivery through the format of elective courses that spanned several weeks.[8]

The broader health professions education literature has a similar gap in AI curriculum development. Recent studies have noted that, despite the rapid uptake of AI application in nursing care, there is a lack of evidence on how to integrate AI education into nursing training and a need for “didactic content on AI use.”[9,10] In a survey study of physical therapy educators, both administrators and students indicated that many of them continue to “harbor reservations and uncertainties” regarding the use of AI.[11]

Notably, there are no current instructional modules freely available on *MedEdPORTAL* or in the broader health professions education literature that provide background on AI, applications of AI in health care and health professions education, case studies of AI being used in clinical care and research, or resources for continuous learning. The American Medical Association recently published a series of modules for health professionals, but access requires membership, and the content is not geared for health professions students.[12] The National Academy of Medicine has made a recommendation that health professions schools should incorporate “core curricula focused on teaching how to appropriately use data science and AI products and services.”[4] We designed an AI module to provide such content to health professions students in an interactive manner, with inclusion of knowledge tests, videos, and links to external resources to remain current.

Methods

Module Content

We developed this introductory AI learning module (Appendix A) to provide foundational AI content for health professions students at our medical school, including medical students, nursing students, and physical therapy students who were in their first year of training. This module was specifically designed as prelearning for any potential in-person workshops, during which students might interact with AI and/or virtual reality tools. The module was developed to be an asynchronous, self-paced online learning resource and intended as a primer for students who had no prior knowledge about these technologies. We developed the module to take no more than 30 minutes of independent study time.

In developing the module, we recruited expertise from multiple disciplines, including instructional design, computer science, engineering, ethics, informatics, nursing, medicine, physical therapy, and our media team. A working group of experts in these areas was convened to perform a literature review and generate educational objectives, content, and assessment methods. The module was designed to have a structure in which the educational objectives and a premodule knowledge test would precede background sections on the use of AI in clinical care and health professions education. After reviewing this background, students would then be able to fully understand the videos that contained case studies. Our media team helped coordinate, capture, and edit videos of health system faculty members who were deeply engaged in AI in their clinical work and research; this included a video depicting a senior ethicist and clinician describing the humanistic and ethical implications of using these tools. The videos would be followed by a postmodule knowledge test and a feedback survey. In addition, to meet our final educational objective of remaining current, our engineering team compiled a list of resources that students could use to stay abreast of the content after completion of the module.

Pre-/Postmodule Test

Our instructional designers assisted with the development of interactive elements and a pre/postmodule knowledge test (Appendix B) that would assess improvement in students’ knowledge of AI-related items that were linked to our educational objectives. There were 10 questions on the pre/postmodule test. The correct response to each question was weighed 5 points, for a total of 50 points, and the overall mean percentage of students with correct responses was determined.

Copyright © 2025 Agarwal et al. This is an open-access publication distributed under the terms of the Creative Commons Attribution license.

Participants and Setting

Once final edits were completed, the module was distributed via our learning management system (LMS) to 200 first-year medical students, who were allowed 3 weeks to complete the module. The module was delivered to medical students as part of a course called “Medicine as a Profession” which covers many objectives related to developing foundational skills of becoming a physician. The module was a required assignment for that course. Completion of the module was tracked by course faculty within our LMS. As this was an independently completed module, no facilitators were required.

Since we planned to share this module with other health professions schools, it was built on the Articulate 360 Platform. This would enable easy transfer of a package to any end user’s LMS and would also allow end users to download the foundational module that could be used as needed as prelearning for their own AI workshops. Appendix A is a folder with the web-based version of the learning module. This format will allow schools to upload the module to their web server (to whatever location they use to host web-based learning content). A file labeled “index.html” will be the starting point for launching the module content and will also allow for sharing the URL required to host the module for public use.

Feedback Survey

A feedback survey was developed and embedded at the end of the module. The survey asked participants to rate the overall quality of the module on a 5-point Likert scale (1 = *poor*; 5 = *excellent*), and to provide responses on the length of time needed to complete the module and any qualitative feedback (Appendix C). The authors reviewed participants’ responses to open-ended questions to identify common themes.

Ethical Approval

This project was reviewed by the University of Miami Miller School of Medicine Institutional Review Board and was deemed to meet the criteria for exemption.

Results

A total of 164 first-year health professions students completed both the pre- and postmodule knowledge tests, and 144 completed the feedback survey, although some did not answer all items of the feedback survey.

Pre-/Postmodule Test

The percentages of students correctly answering the 10 questions on the pre- and postmodule knowledge test are indicated in the Table. The mean percentage of students correctly answering the premodule test items was 74%, compared to 87% of students answering correctly on the postmodule test (two-tailed $p < .001$). This increase from pre- to postmodule illustrates that the module had a statistically significant positive impact on student knowledge base. Questions 4 and 7 had the lowest percentage of students with correct answers on both the pre- and postmodule tests. Question 4 asked students about the use of AI in enhancing virtual reality environments, and Question 7 focused on applications of AI in billing and reimbursement. Questions 8 and 10 had the highest percentage of students with correct answers on both the pre- and postmodule tests; these two questions focused on applications of AI in electronic health records and health care administration.

Feedback Survey

In the first question of the feedback survey, students were asked to rate the overall quality of the AI in Medicine module on a 5-point Likert scale (Figure). A total of 129 (96%) of 135 students

Table. Pre- and Postmodule Knowledge Test Results ($N = 164$)

Question	Pre % Correct	Post % Correct
What is Machine Learning (ML)?	69	82
Which of the following is an example of AI not using machine learning?	66	82
What is Generative AI (GenAI) primarily used for?	79	94
How does Artificial Intelligence (AI) enhance the experience in Virtual Reality (VR) environments?	44	66
In Augmented Reality (AR), what role does AI play?	72	91
What is a key feature of Mixed Reality (MR) that differentiates it from AR and VR?	88	98
Which of the following is an example of an application of AI in healthcare?	61	74
What is a major application of AI in electronic health records (EHRs)?	91	98
A primary care clinic employs an AI tool for remote patient monitoring in chronic disease management. The main purpose of this AI tool is to:	84	92
In a healthcare system, AI is used to streamline the reimbursement process. This AI application is primarily focused on:	89	95
M Overall Total	74[a]	87[b]

Abbreviations: AI, artificial intelligence; AR, augmented reality; EHR, electronic health records; GenAI, generative AI; ML, machine learning; MR, mixed reality; VR, virtual reality.
[a] $SD = 15$.
[b] $SD = 11$.

Copyright © 2025 Agarwal et al. This is an open-access publication distributed under the terms of the Creative Commons Attribution license.

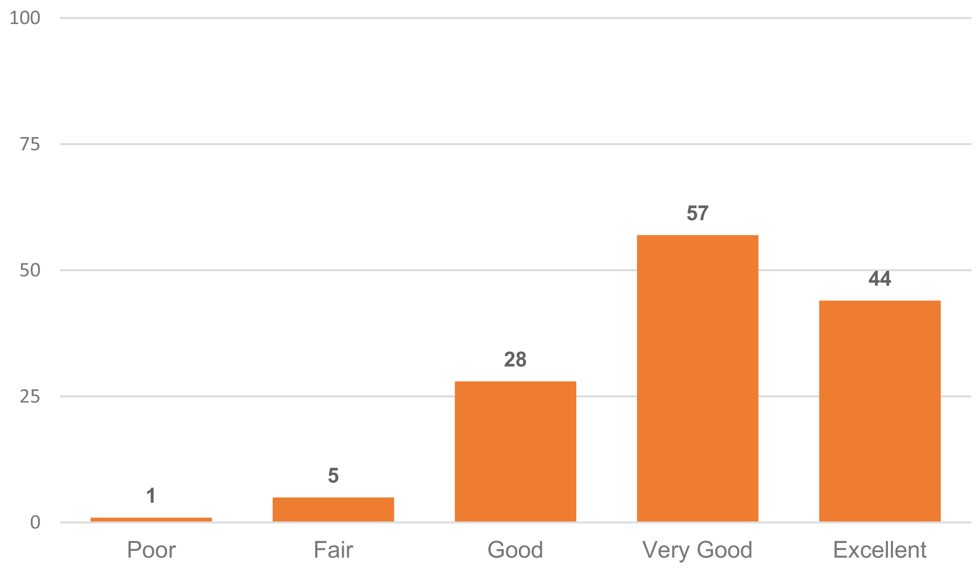

Figure. Percentages of health professions students (N = 135) rating the quality of the introductory artificial intelligence (AI) learning module in response to the feedback survey question: Please rate the overall quality of the AI in Medicine module on a scale from 1 to 5, with 1 being *poor* and 5 being *excellent*.

who answered this question rated the module as *good*, *very good*, or *excellent*. A total of 92 respondents (69%) reported that they completed the module in 15-30 minutes, compared to 28 respondents (21%) who indicated that they needed more than 30 minutes, and 14 respondents (11%) who reported taking fewer than 15 minutes to complete the module. Overall, most participants found the module to be manageable within a 15- to 30-minute time frame.

The majority of participants, 108 (91%) of 119, felt that the module has clear applicability to their future careers, highlighting its relevance and potential impact on their professional development. Three participants recognized the potential future relevance, though they did not find it immediately applicable.

Feedback Survey Comments

Many participants felt that the most valuable and interesting aspect of the module was its inclusion of short videos featuring real-world examples of AI applications, particularly those involving physicians from different specialties. These videos were praised for their clarity, brevity, and ability to provide concrete, practical insights into how AI is currently being used in various medical fields. The specific clinical applications and the perspectives of faculty were highlighted as particularly engaging and informative. The organization of the module, including the clear definitions of key concepts and terms, was valued for making the content user-friendly and easy to follow. Common areas of confusion included the distinctions between various forms of reality (augmented reality, mixed reality, and virtual reality) and the differences between machine learning and other types of AI. A few respondents suggested that more video content and detailed explanations might be provided to help clarify these concepts. Additionally, the ethical aspects of AI and specific processes, such as reimbursement, were mentioned as areas where more information would be beneficial. A few students asked for more advanced content for those already familiar with AI. Specific suggestions included making the module more interactive by breaking up text-heavy sections and addressing ethical concerns more thoroughly. Participants also recommended providing captions for videos to improve accessibility. Overall, although the module was well-received, these suggestions highlight areas for potential enhancement to better meet the needs of all health professions students.

Discussion

This module on AI for health professions students was designed to fill a gap in curricula available to health professions schools nationally, particularly for those schools who may not have access to expertise within the disciplines utilized to develop this module. We convened the requisite experts and tasked them with designing and providing content for this module. It can be used as prelearning for any in-person session on AI or as a stand-alone learning resource.

As this is a rapidly evolving field, we wanted to ensure that learners had foundational elements that were unlikely to change

Copyright © 2025 Agarwal et al. This is an open-access publication distributed under the terms of the Creative Commons Attribution license.

(e.g., definitions of machine learning and virtual reality) as well as a current list of resources to maintain their connection to the field and stay abreast of continued developments. We also wanted to ensure that the module was not time-consuming for busy health care students by creating a module that is easy to complete within 15-30 minutes.

Learners in our pilot study demonstrated a significant increase in their knowledge base and provided positive feedback on the module's design and content. Some questions had high percentages of correct responses among students even before exposure to the module, perhaps indicating that students may have already had prior background in these areas of application of AI in electronic health records and health care administration. Questions related to virtual reality and health care reimbursement were the most challenging for our learners. Consequently, we worked on improving the clarity of the sections on virtual reality, ethics, and billing processes. Overall, the premodule test identified areas of weakness, and all questions had improved performance after exposure to the module. The design of a short pre- and postmodule test allows students to assess their individual areas of weakness and assess their progression in knowledge at the completion of the module. Among the feedback received, some comments suggested breaking apart text-heavy sections, including adding more questions, and including more video-based content that is captioned. We made edits to the module to include more interactive and reflective questions. With the LMS package, schools can utilize the content for their local needs and use it as prelearning for their own interactive in-person workshops on AI and virtual reality. Since the time of the initial pilot, we have added one video about the use of AI in physical therapy.

At our school, we used this module prior to an in-person 4-hour workshop in which medical students used a variety of large language models, image generators, and virtual reality headsets and discussed the benefits and limitations of these tools with faculty. The workshop also included art observation and art creation activities followed by narrative reflective questions that asked students about their ideas on the future integration of these tools and the essential human skills that they did not think could be replaced by AI. Other health professions students at our school (nursing and physical therapy) were not enrolled in courses during the time of this pilot, but we have included them in subsequent iterations of our workshop as the content is applicable to all health professions students who are early in their training and does not require further modification for those learners.

We encourage schools to use the content of this introductory AI learning module as a starting point for their school's own content. Schools that use this module as prelearning prior to an in-person workshop can consider linking it to existing ethics and humanities small-group sessions and use the narrative reflection questions at the end of the module as a starting point for discussion. We have embedded a PDF form into the module that contains downloadable questions that students may respond to by writing down their narrative reflections and then sharing them with faculty. Access to the module was provided at our school 1 month in advance of the in-person workshop and performance on the module tests was provided as formative feedback.

A limitation of the module is that technology is rapidly evolving and some of the content may need to be revised. However, we have added resources within the references for students to remain current on advances in technology. While the videos in the module are drawn from a single institution, they illustrate use cases of this technology which are broadly applicable and not specific to our medical school. We believe that other institutions without direct access to experts in computer science, engineering, ethics, and health informatics would find this content particularly valuable. In addition, although the content was intended and developed for a larger population of health professions students, including medical, nursing, and physical therapy students, the pilot study and survey feedback was exclusively within a population of first-year medical students.

We hope this module is helpful to health professions students in introducing them to the rapidly emerging applications of AI in health care and allowing them to reflect on their use of these tools in their future practice.

Appendices

A. AI in Medicine folder

B. Pre- and Posttest.docx

C. Feedback Survey.docx

All appendices are peer reviewed as integral parts of the Original Publication.

Gauri Agarwal, MD: Associate Professor of Medicine and Medical Education, Associate Professor of Informatics and Health Data Sciences, Department of Medicine, Associate Dean for Curriculum, University of Miami Miller School of Medicine; ORCID: https://orcid.org/0000-0001-9786-5948

Copyright © 2025 Agarwal et al. This is an open-access publication distributed under the terms of the Creative Commons Attribution license.

Lokesh Ramamoorthi, MS, MBA: Lecturer of Software Engineering, Department of Electrical and Computer Engineering; Program Co-Director, Bachelors Program in Innovation, Technology, and Design, University of Miami

Trevor Yuen, MS: Executive Director, Data Science and Engineering, UHealth Information Technology, University of Miami

Edwin Merced, EdD: Senior Instructional Designer, University of Miami Miller School of Medicine

Jacqueline Brenner: Third-Year Medical Student, University of Miami Miller School of Medicine

Winfred Wu, MD, MPH: Executive Director, Research Informatics, UHealth Information Technology, University of Miami; Director, Health Informatics, Miami Clinical and Translational Science Institute, Volunteer Assistant Professor, Department of Medicine, University of Miami Miller School of Medicine

Richard Sabina, PhD: Adjunct Professor, Department of Foundational Medical Studies, Oakland University William Beaumont School of Medicine; *MedEdPORTAL* Faculty Mentor

Acknowledgments
The authors wish to acknowledge the *MedEdPORTAL* Scholar Development Course as a key developmental resource for disseminating this educational activity.

Disclosures
None to report.

Funding/Support
None to report.

Prior Presentations
Agarwal G. The HUMANN Project: the humanities underpinning machines and artificial neural networks. Poster presented at: Harvard Macy Institute Program for Educators in Health Professions; May 2024; Boston, MA.

Ethical Approval
University of Miami Miller School of Medicine Institutional Review Board reviewed this project.

References

1. Frenk J, Chen LC, Chandran L, et al. Challenges and opportunities for educating health professionals after the COVID-19 pandemic. *Lancet.* 2022;400(10362):1539-1556. https://doi.org/10.1016/S0140-6736(22)02092-X
2. Alrassi J, Katsufrakis PJ, Chandran L. Technology can augment, but not replace, critical human skills needed for patient care. *Acad Med.* 2021;96(1):37-43. https://doi.org/10.1097/ACM.0000000000003733
3. Topol EJ. High-performance medicine: the convergence of human and artificial intelligence. *Nat Med.* 2019;25(1):44-56. https://doi.org/10.1038/s41591-018-0300-7
4. National Academy of Medicine; The Learning Health System Series; Whicher D, Ahmed M, Thadaney Israni S, Matheny M, eds. Artificial Intelligence in Health Care: The Hope, the Hype, the Promise, the Peril. National Academies Press (US); 2023. https://doi.org/10.17226/27111
5. Sahni NR, Carrus B. Artificial intelligence in U.S. health care delivery. *N Engl J Med*. 2023;389(4):348-358. https://doi.org/10.1056/nejmra2204673
6. Topol EJ. *Deep Medicine: How Artificial Intelligence Can Make Health Care Human Again*. Aspen Ideas. 2019. Accessed November 17, 2024. https://www.aspenideas.org/sessions/deep-medicine-how-artificial-intelligence-can-make-health-care-human-again
7. Lee J, Wu AS, Li D, Kulasegaram KM. Artificial intelligence in undergraduate medical education: a scoping review. *Acad Med.* 2021;96(11S):S62-S70. https://doi.org/10.1097/ACM.0000000000004291
8. Gordon M, Daniel M, Ajiboye A, et al. A scoping review of artificial intelligence in medical education: BEME Guide No. 84. *Med Teach.* 2024;46(4):446-470. https://doi.org/10.1080/0142159X.2024.2314198
9. Montejo L, Fenton A, Davis G. Artificial intelligence (AI) applications in healthcare and considerations for nursing education. *Nurse Educ Pract.* 2024;80:104158. https://doi.org/10.1016/j.nepr.2024.104158
10. Lifshits I, Rosenberg D. Artificial intelligence in nursing education: a scoping review. *Nurse Educ Pract*. 2024;80:104148. https://doi.org/10.1016/j.nepr.2024.104148
11. Severin R, Gagnon K. An early snapshot of attitudes toward generative artificial intelligence in physical therapy education. *J Phys Ther Educ*. 2024. https://doi.org/10.1097/JTE.0000000000000381
12. Artificial intelligence. *AMA Ed Hub.* Accessed April 4, 2025. https://edhub.ama-assn.org/collections/44024/artificial-intelligence

Received: August 28, 2024
Accepted: March 31, 2025
Published: May 1, 2025

Copyright © 2025 Agarwal et al. This is an open-access publication distributed under the terms of the Creative Commons Attribution license.
