# Supplementary figures and images for: Exploring Applications of Artificial Intelligence Tools in Clinical Care and Health Professions Education: An Online Module for Students
Source: MedEdPORTAL. 2025 May 1;21:11524. doi: 10.15766/mep_2374-8265.11524 (PMC12043951; doi:10.15766/mep_2374-8265.11524)

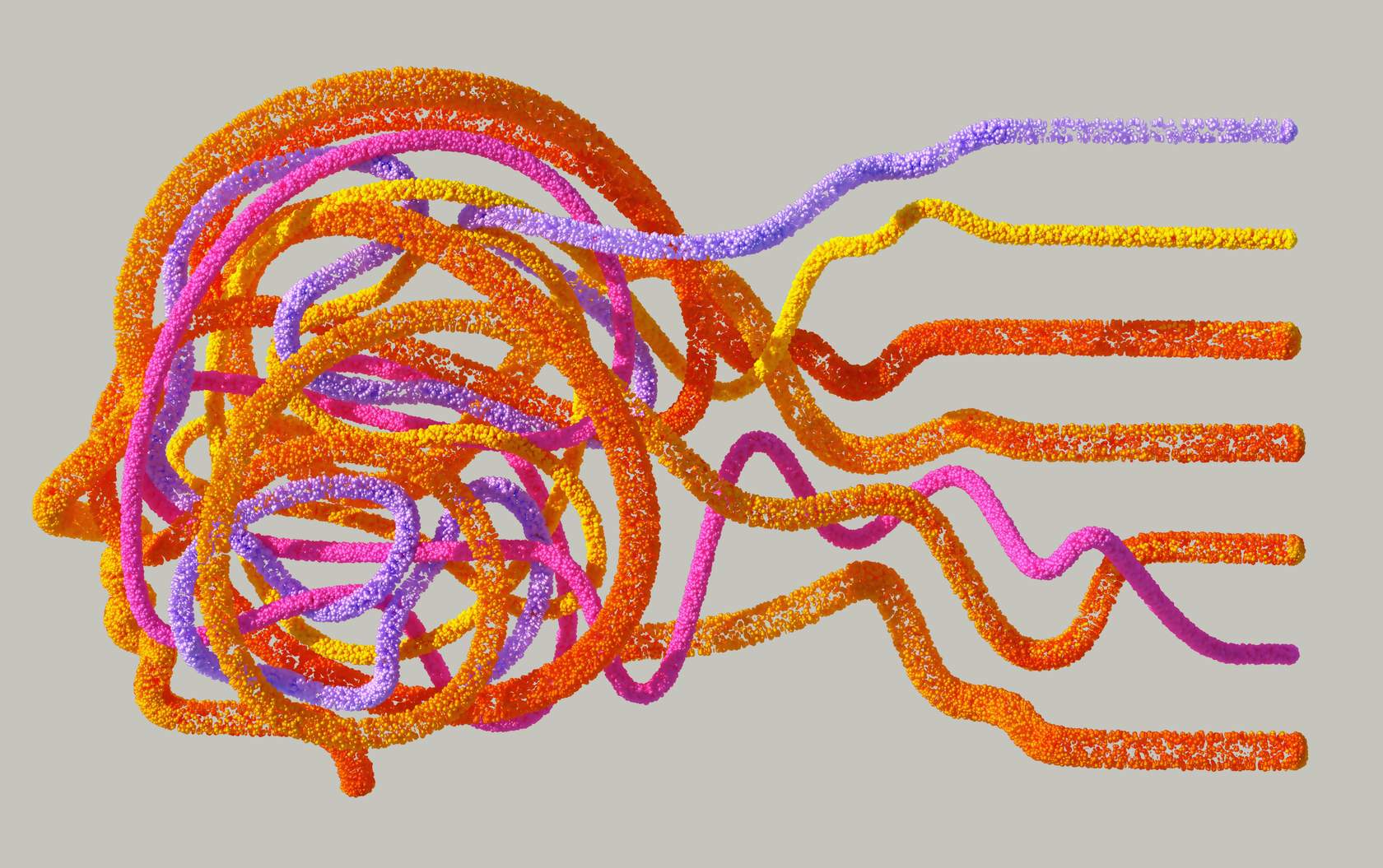

Supplement: Supplementary file 1 — AI in Medicine folderPre- and Posttest.docxFeedback Survey.docx [file mep_2374-8265.11524-s001.zip › A. AI in Medicine/assets/0bDika/stock-image.jpg]

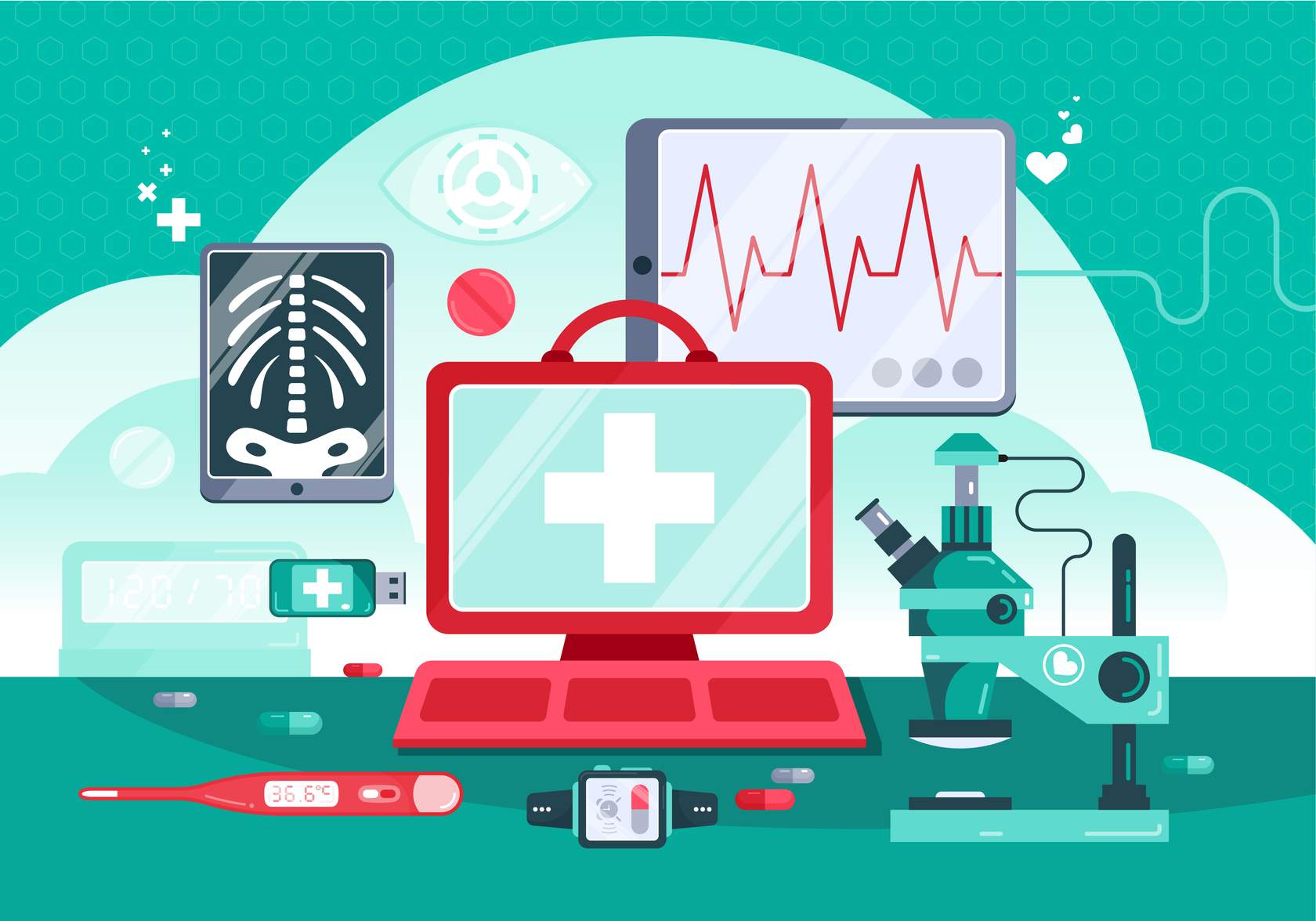

Supplement: Supplementary file 1 — AI in Medicine folderPre- and Posttest.docxFeedback Survey.docx [file mep_2374-8265.11524-s001.zip › A. AI in Medicine/assets/2003.i518.003_digital medicine illustration.jpg]

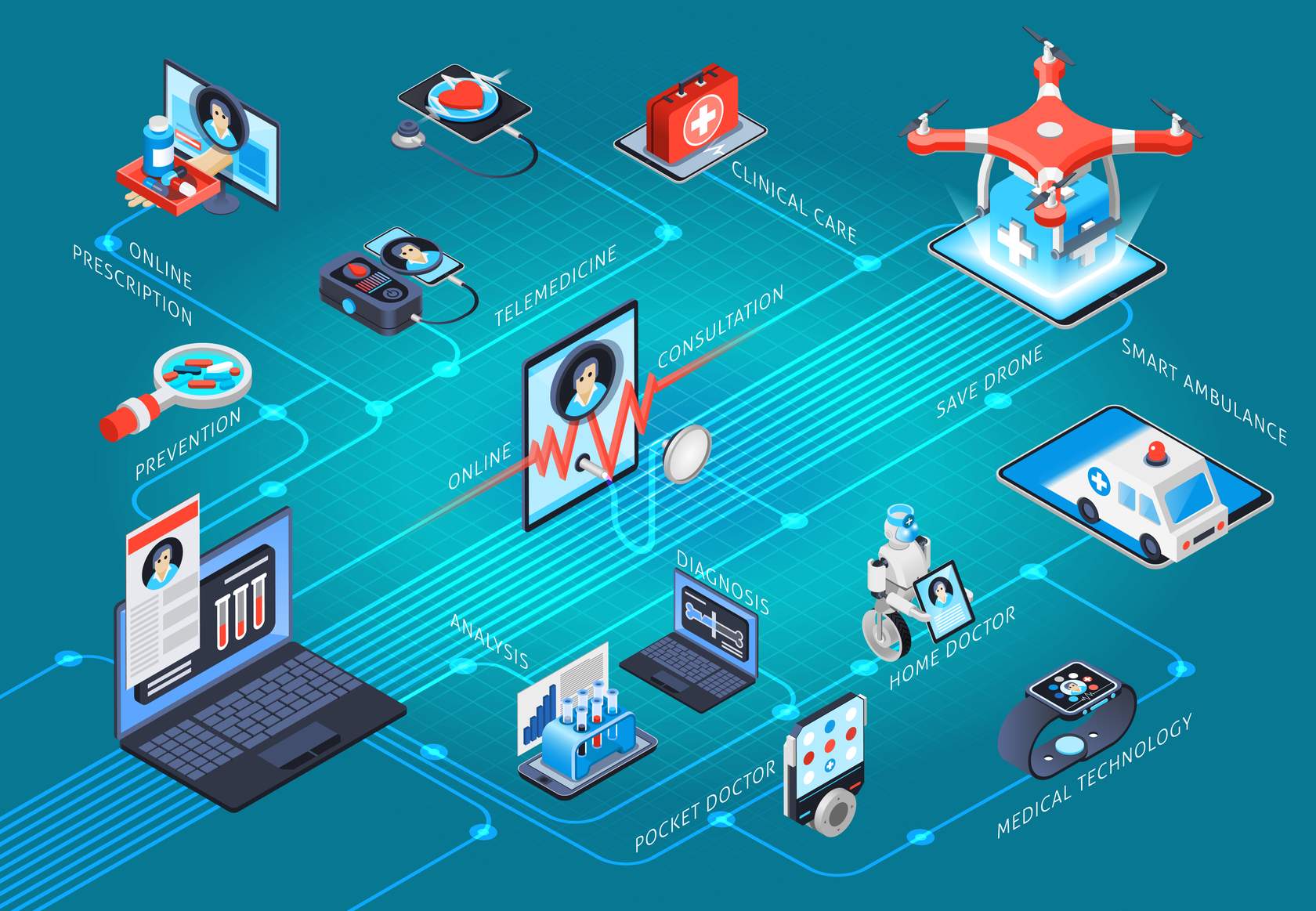

Supplement: Supplementary file 1 — AI in Medicine folderPre- and Posttest.docxFeedback Survey.docx [file mep_2374-8265.11524-s001.zip › A. AI in Medicine/assets/24966.jpg]

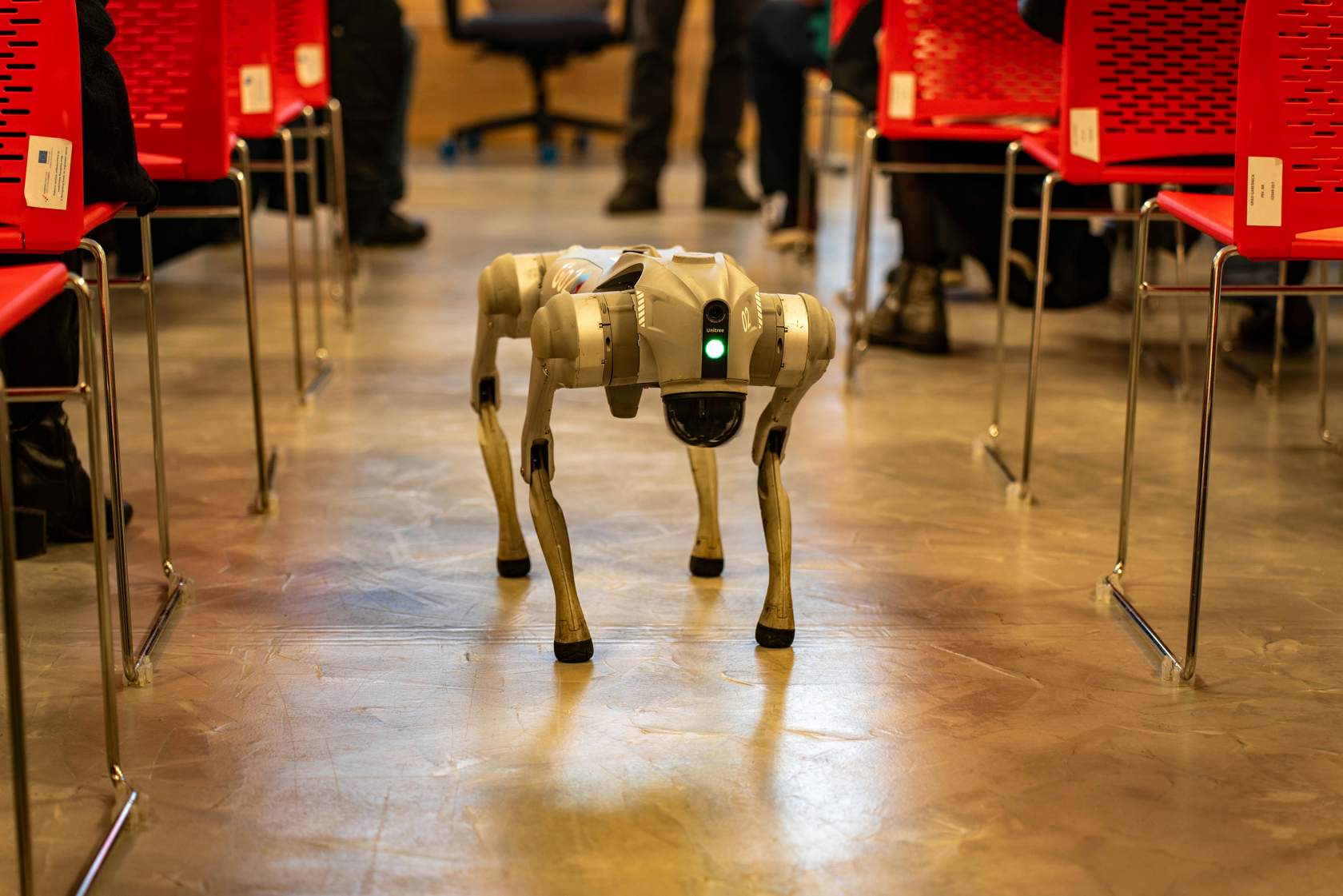

Supplement: Supplementary file 1 — AI in Medicine folderPre- and Posttest.docxFeedback Survey.docx [file mep_2374-8265.11524-s001.zip › A. AI in Medicine/assets/5G5baJ/stock-image.jpg]

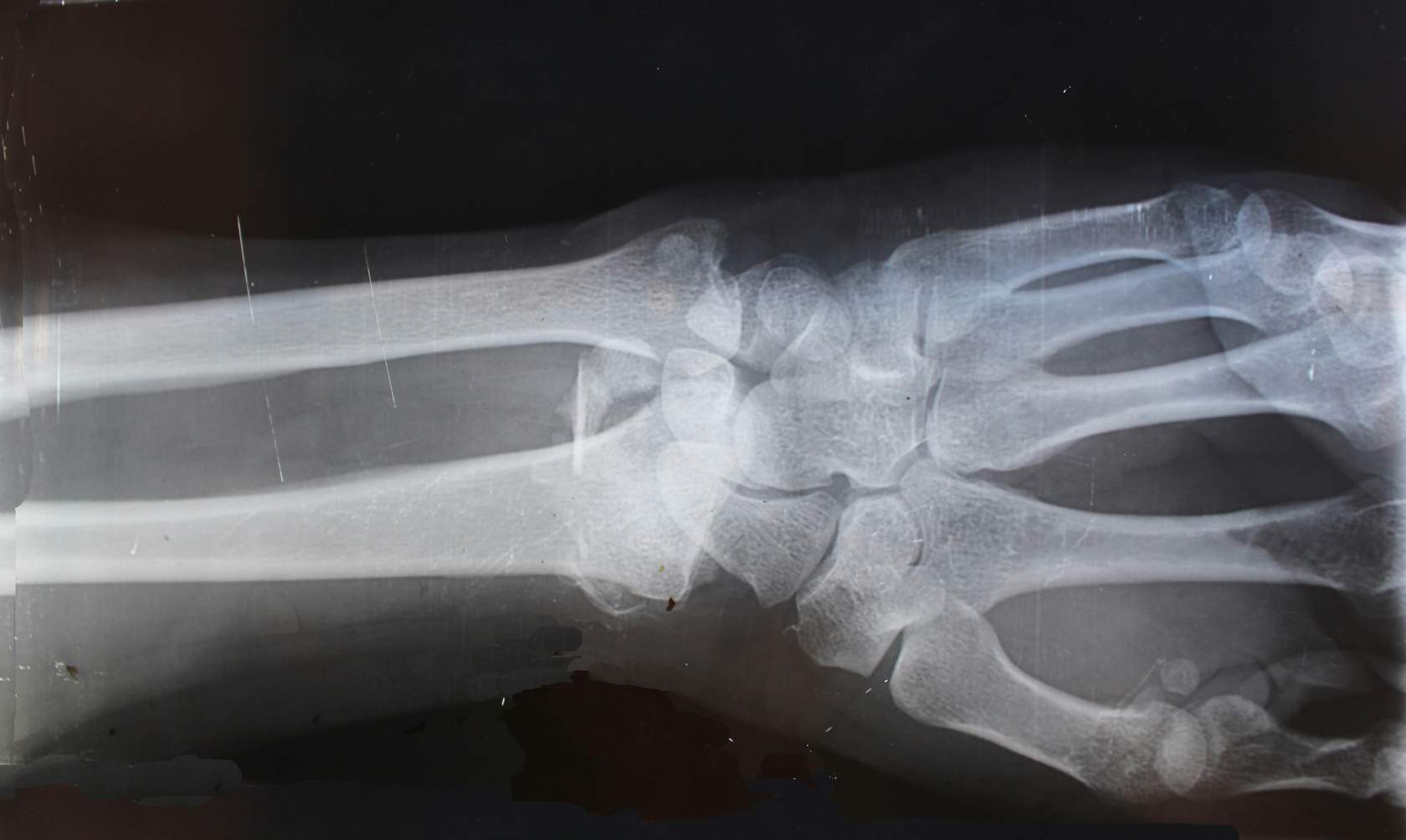

Supplement: Supplementary file 1 — AI in Medicine folderPre- and Posttest.docxFeedback Survey.docx [file mep_2374-8265.11524-s001.zip › A. AI in Medicine/assets/9_svK-/stock-image.jpg]

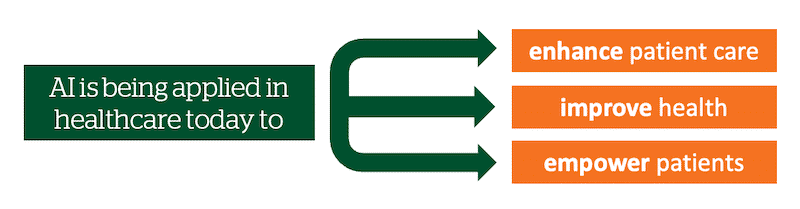

Supplement: Supplementary file 1 — AI in Medicine folderPre- and Posttest.docxFeedback Survey.docx [file mep_2374-8265.11524-s001.zip › A. AI in Medicine/assets/A.png]

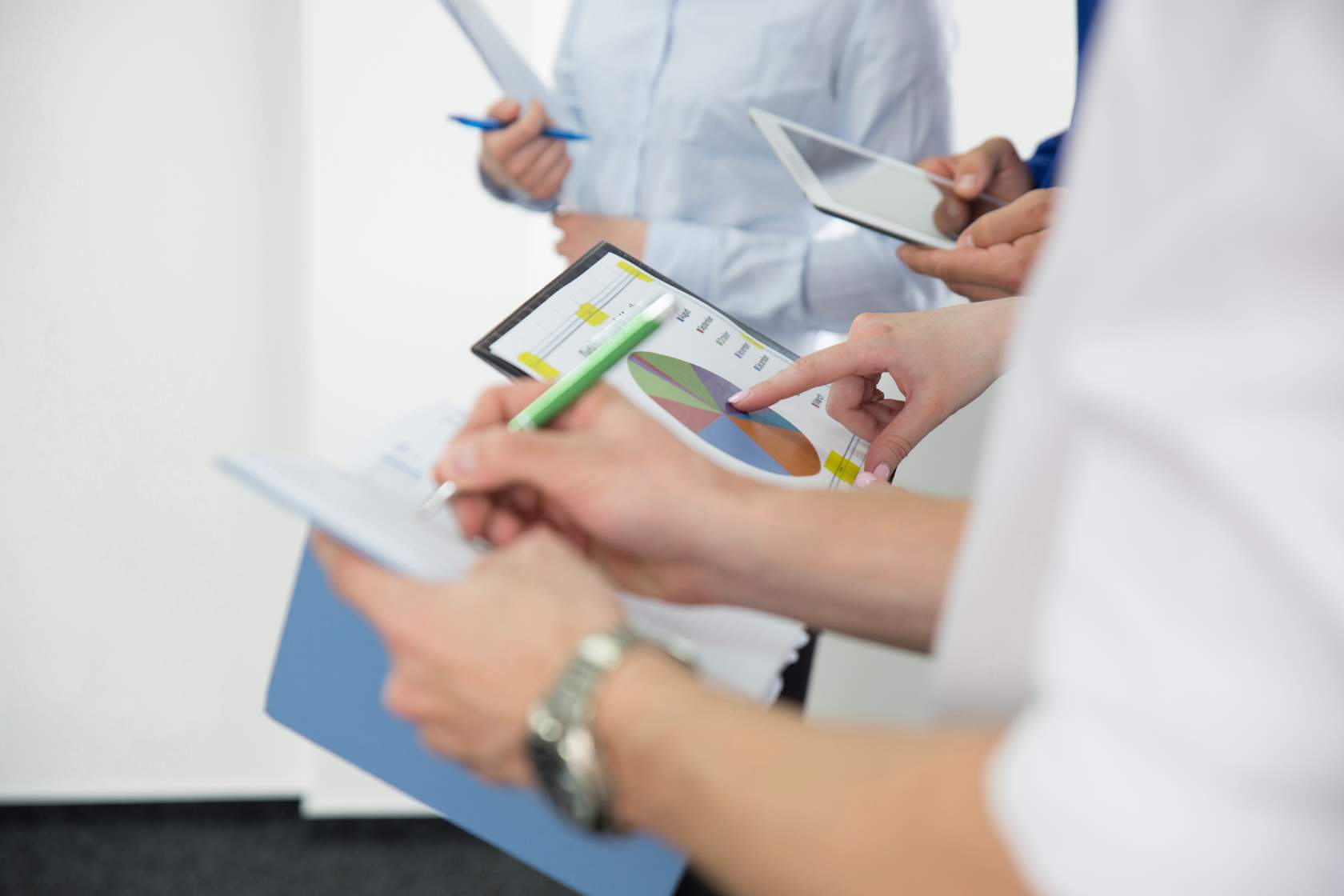

Supplement: Supplementary file 1 — AI in Medicine folderPre- and Posttest.docxFeedback Survey.docx [file mep_2374-8265.11524-s001.zip › A. AI in Medicine/assets/anonymous-coworkers-with-papers.jpg]

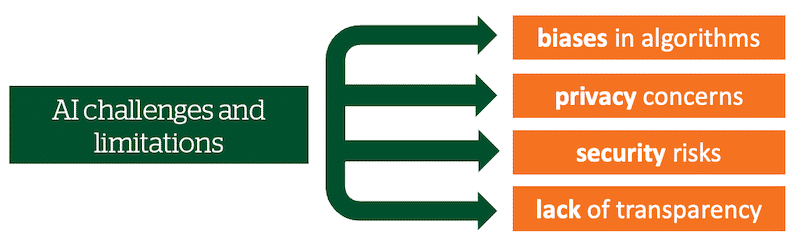

Supplement: Supplementary file 1 — AI in Medicine folderPre- and Posttest.docxFeedback Survey.docx [file mep_2374-8265.11524-s001.zip › A. AI in Medicine/assets/B.png]

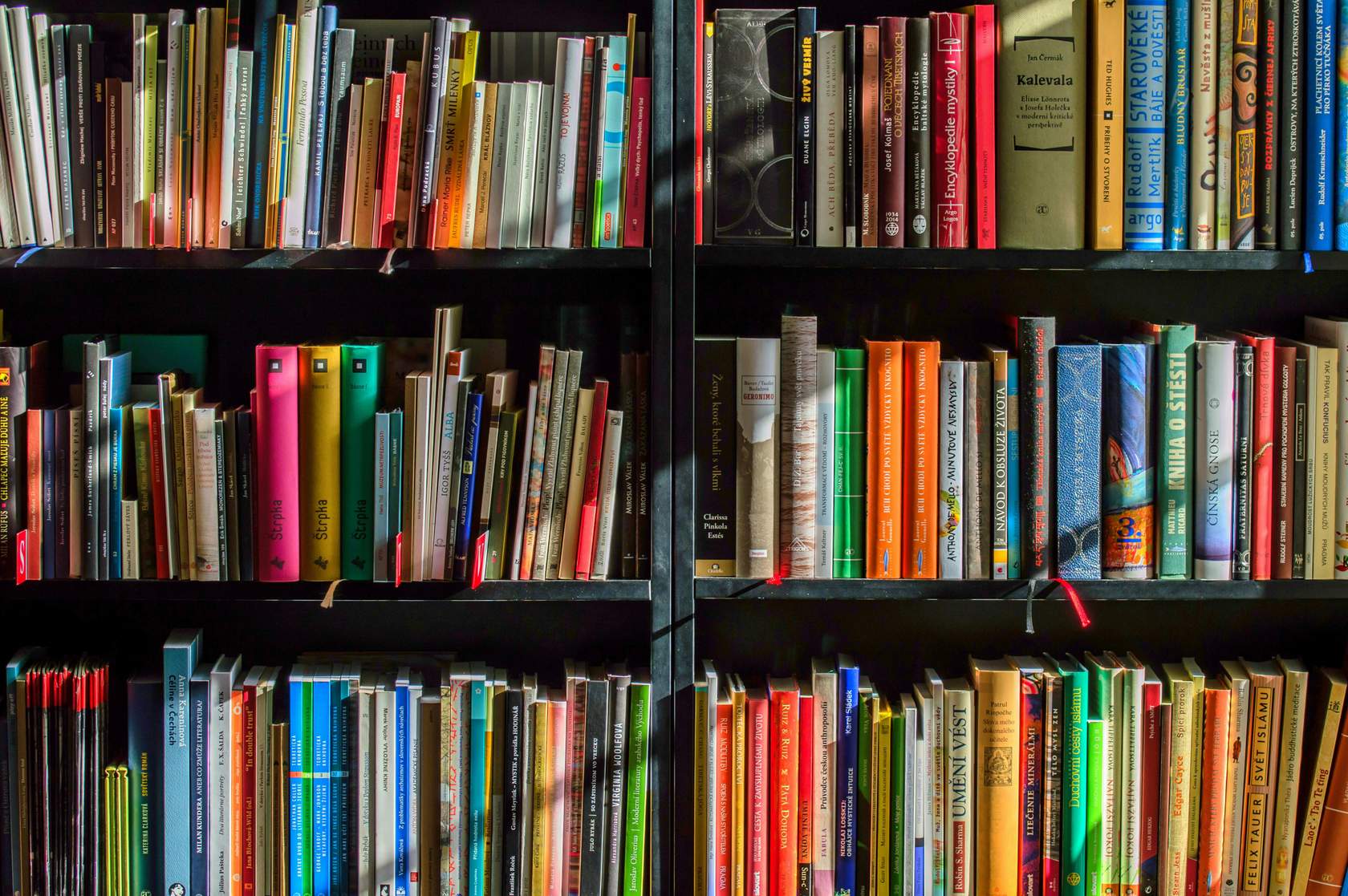

Supplement: Supplementary file 1 — AI in Medicine folderPre- and Posttest.docxFeedback Survey.docx [file mep_2374-8265.11524-s001.zip › A. AI in Medicine/assets/BK9URZ/stock-image.jpg]

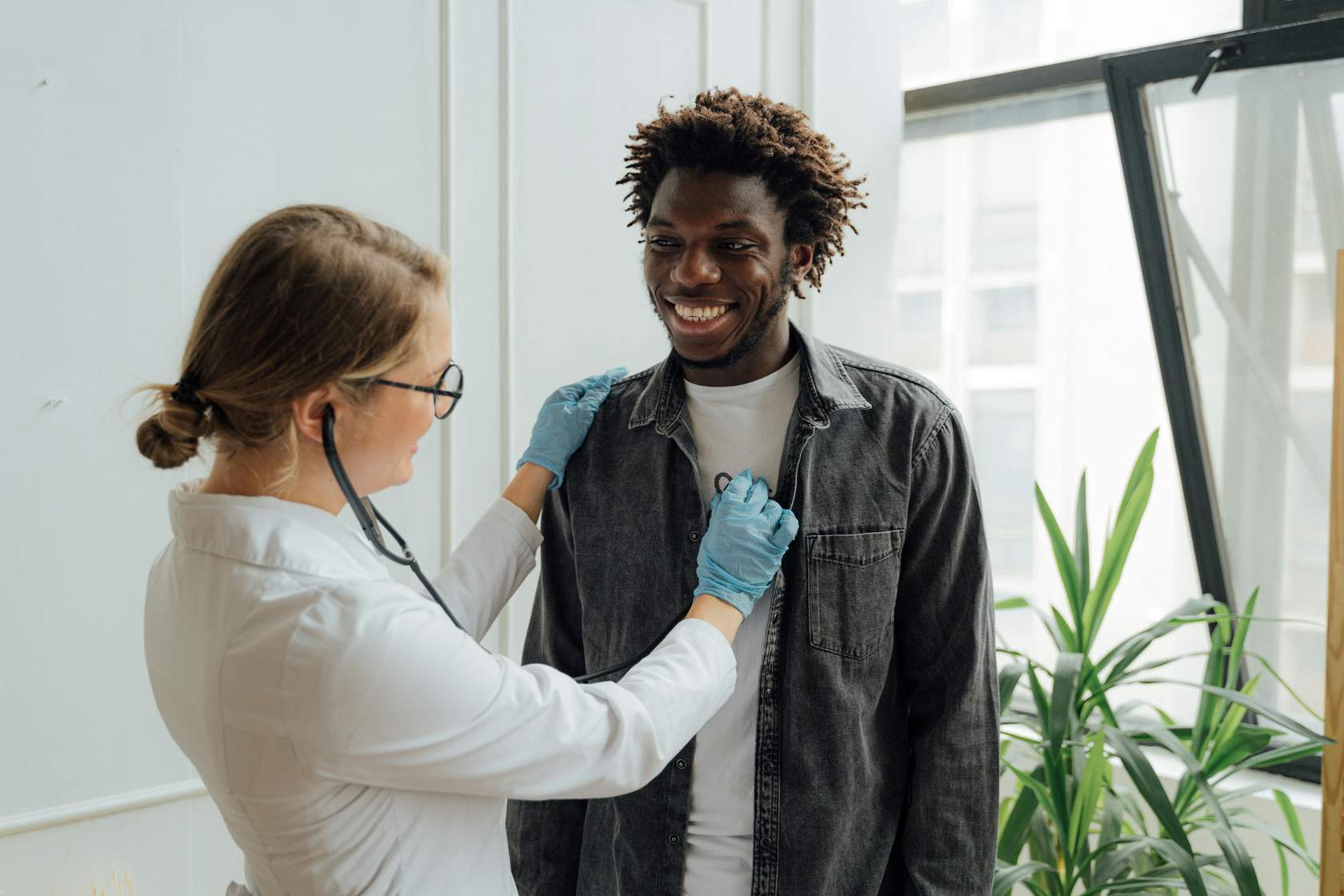

Supplement: Supplementary file 1 — AI in Medicine folderPre- and Posttest.docxFeedback Survey.docx [file mep_2374-8265.11524-s001.zip › A. AI in Medicine/assets/Cd6YQd/stock-image.jpg]

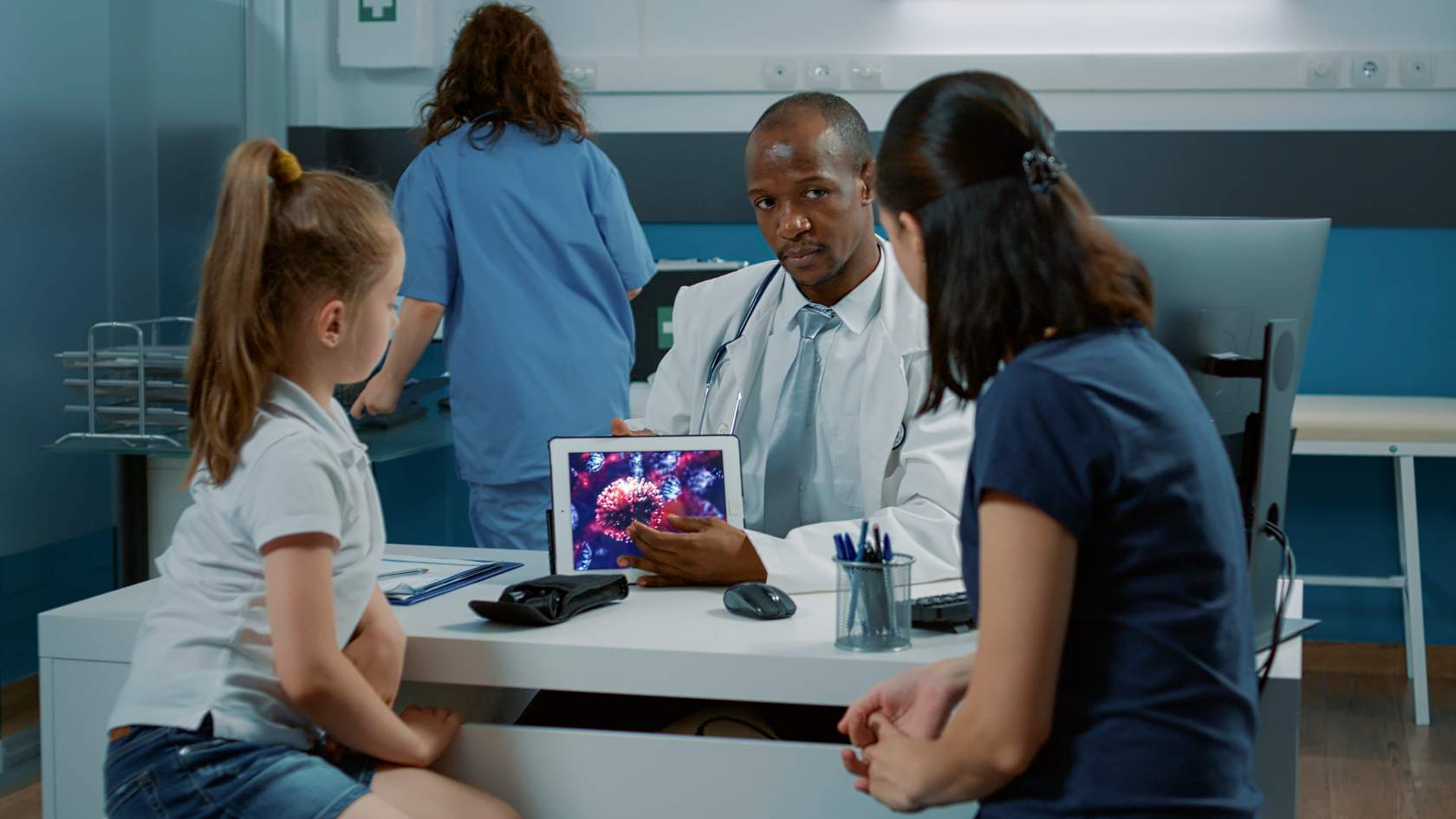

Supplement: Supplementary file 1 — AI in Medicine folderPre- and Posttest.docxFeedback Survey.docx [file mep_2374-8265.11524-s001.zip › A. AI in Medicine/assets/doctor-explaining-coronavirus-illustration-tablet-display.jpg]

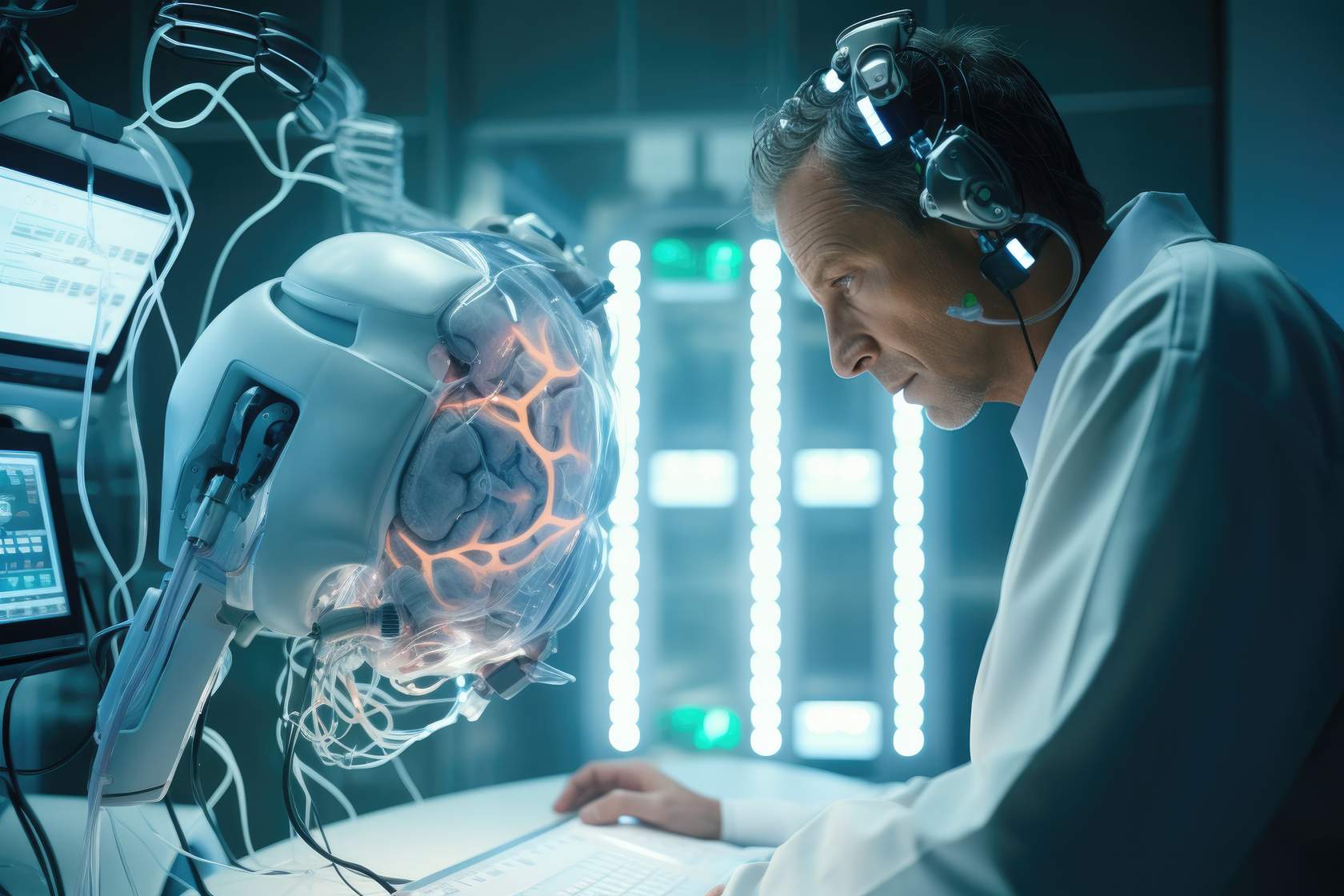

Supplement: Supplementary file 1 — AI in Medicine folderPre- and Posttest.docxFeedback Survey.docx [file mep_2374-8265.11524-s001.zip › A. AI in Medicine/assets/doctor-from-future-concept (1).jpg]

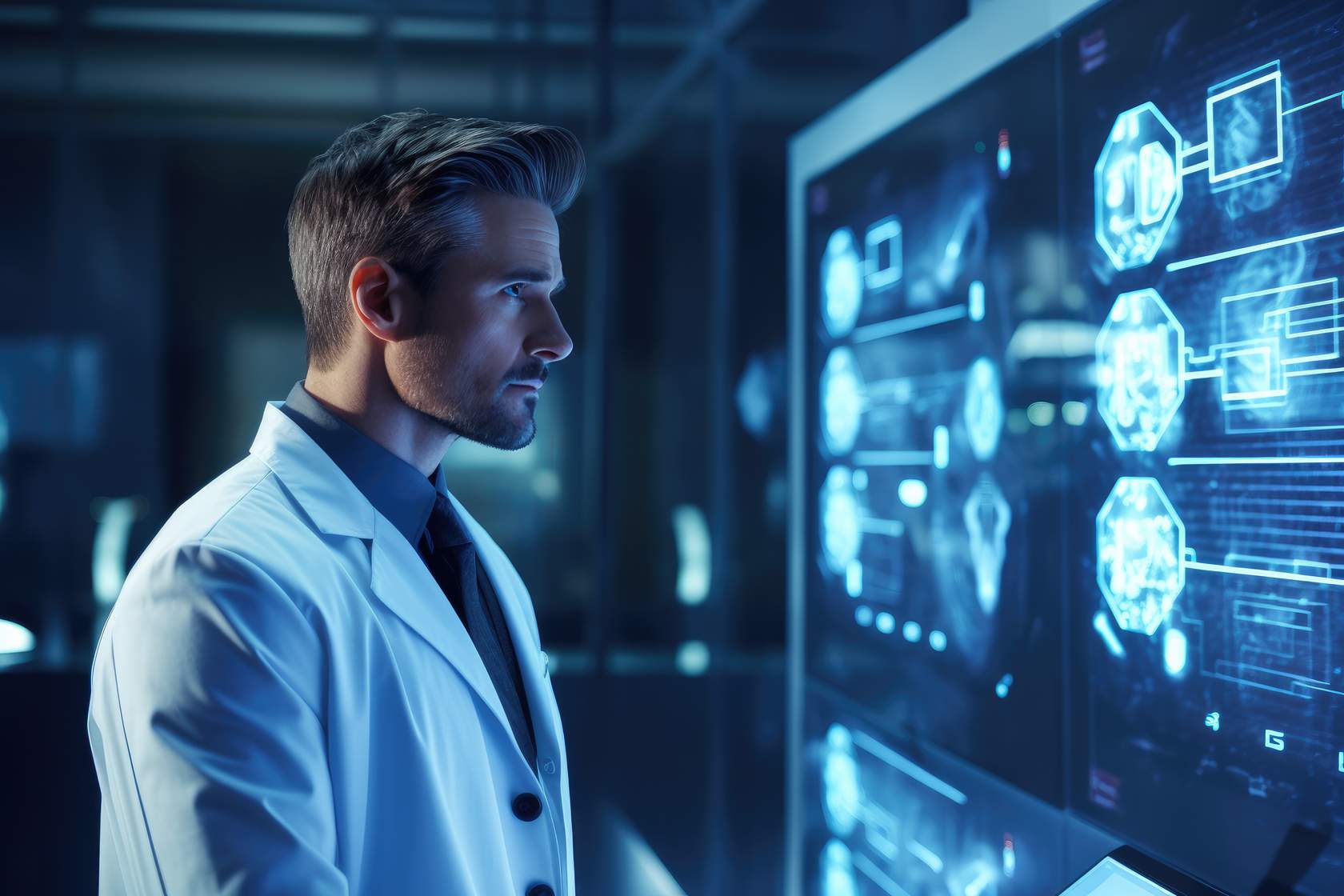

Supplement: Supplementary file 1 — AI in Medicine folderPre- and Posttest.docxFeedback Survey.docx [file mep_2374-8265.11524-s001.zip › A. AI in Medicine/assets/doctor-from-future-concept.jpg]

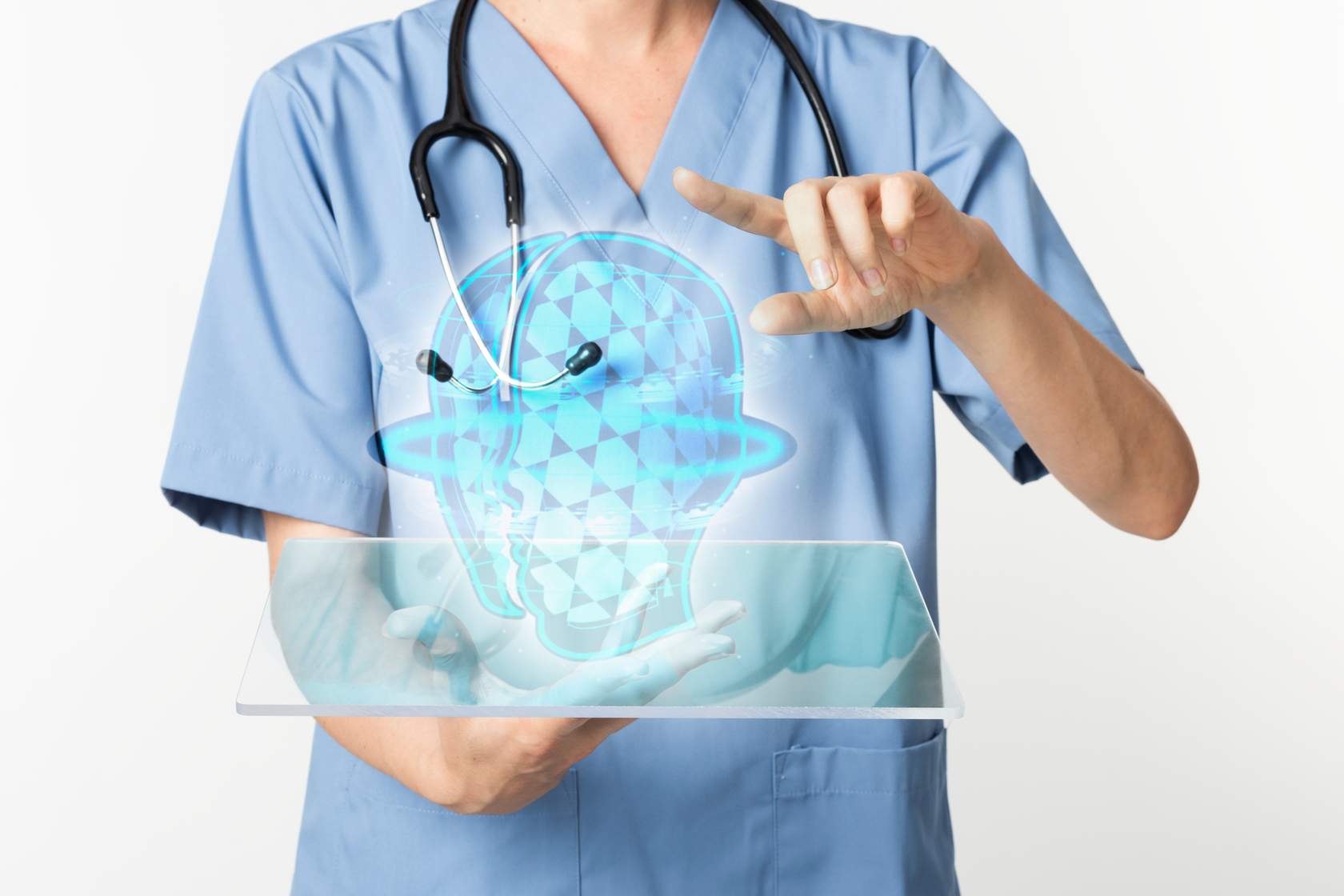

Supplement: Supplementary file 1 — AI in Medicine folderPre- and Posttest.docxFeedback Survey.docx [file mep_2374-8265.11524-s001.zip › A. AI in Medicine/assets/doctors-using-transparent-tablet-with-hologram-medical.jpg]

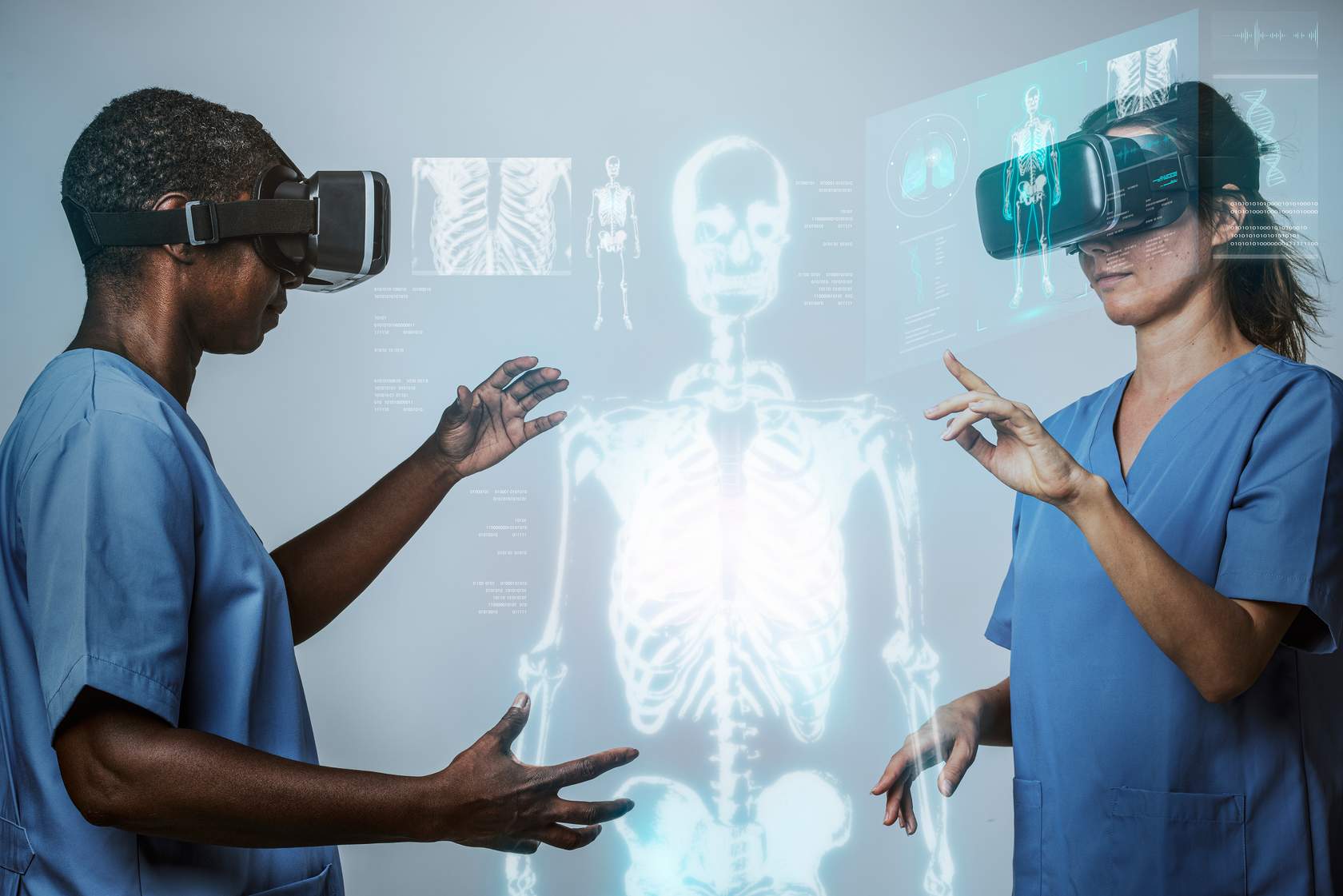

Supplement: Supplementary file 1 — AI in Medicine folderPre- and Posttest.docxFeedback Survey.docx [file mep_2374-8265.11524-s001.zip › A. AI in Medicine/assets/doctors-wearing-vr-simulation-with-hologram-medical.jpg]

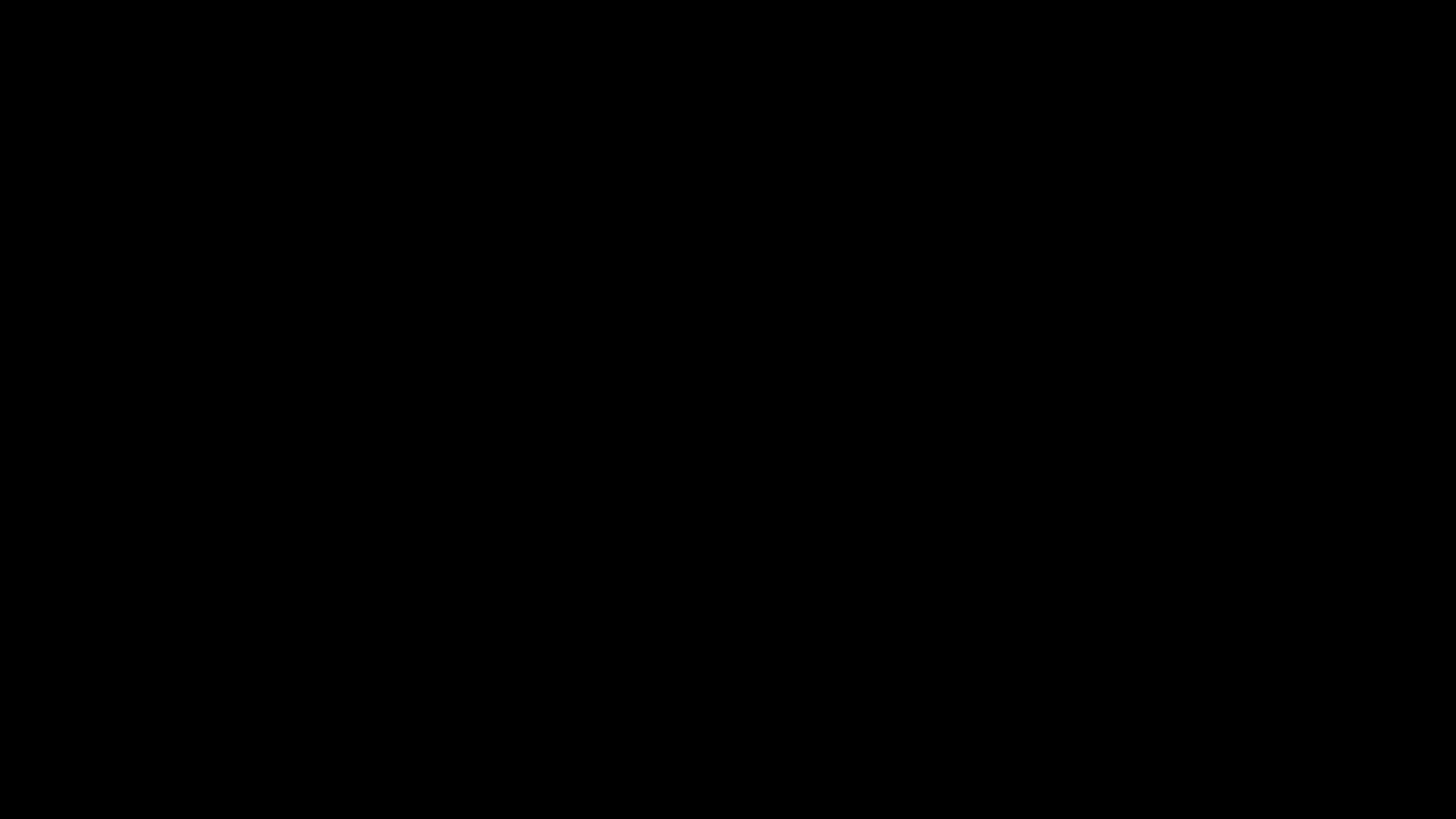

Supplement: Supplementary file 1 — AI in Medicine folderPre- and Posttest.docxFeedback Survey.docx [file mep_2374-8265.11524-s001.zip › A. AI in Medicine/assets/Dr. Agarwal Intro.jpg]

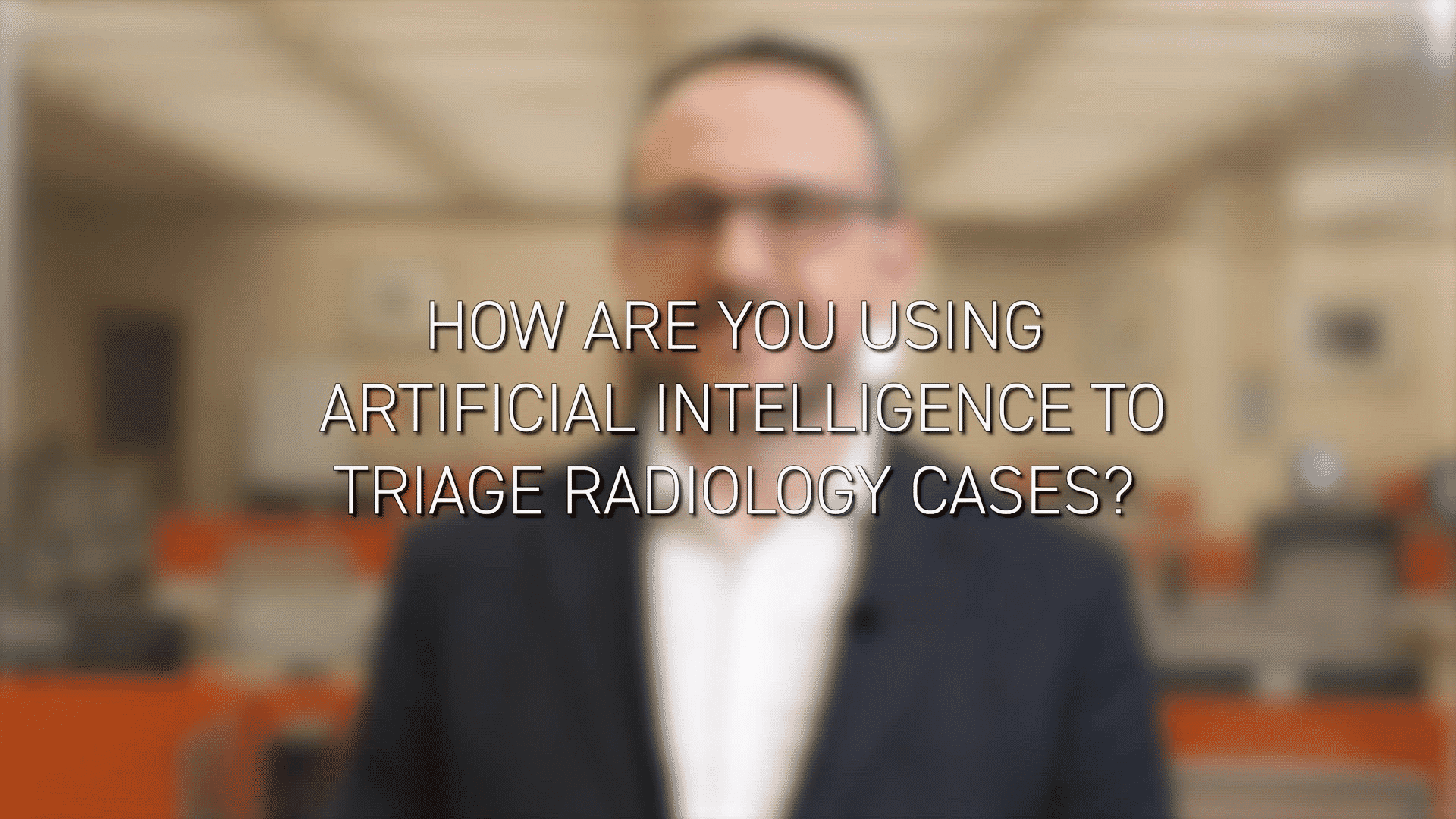

Supplement: Supplementary file 1 — AI in Medicine folderPre- and Posttest.docxFeedback Survey.docx [file mep_2374-8265.11524-s001.zip › A. AI in Medicine/assets/Dr. Braga.jpg]

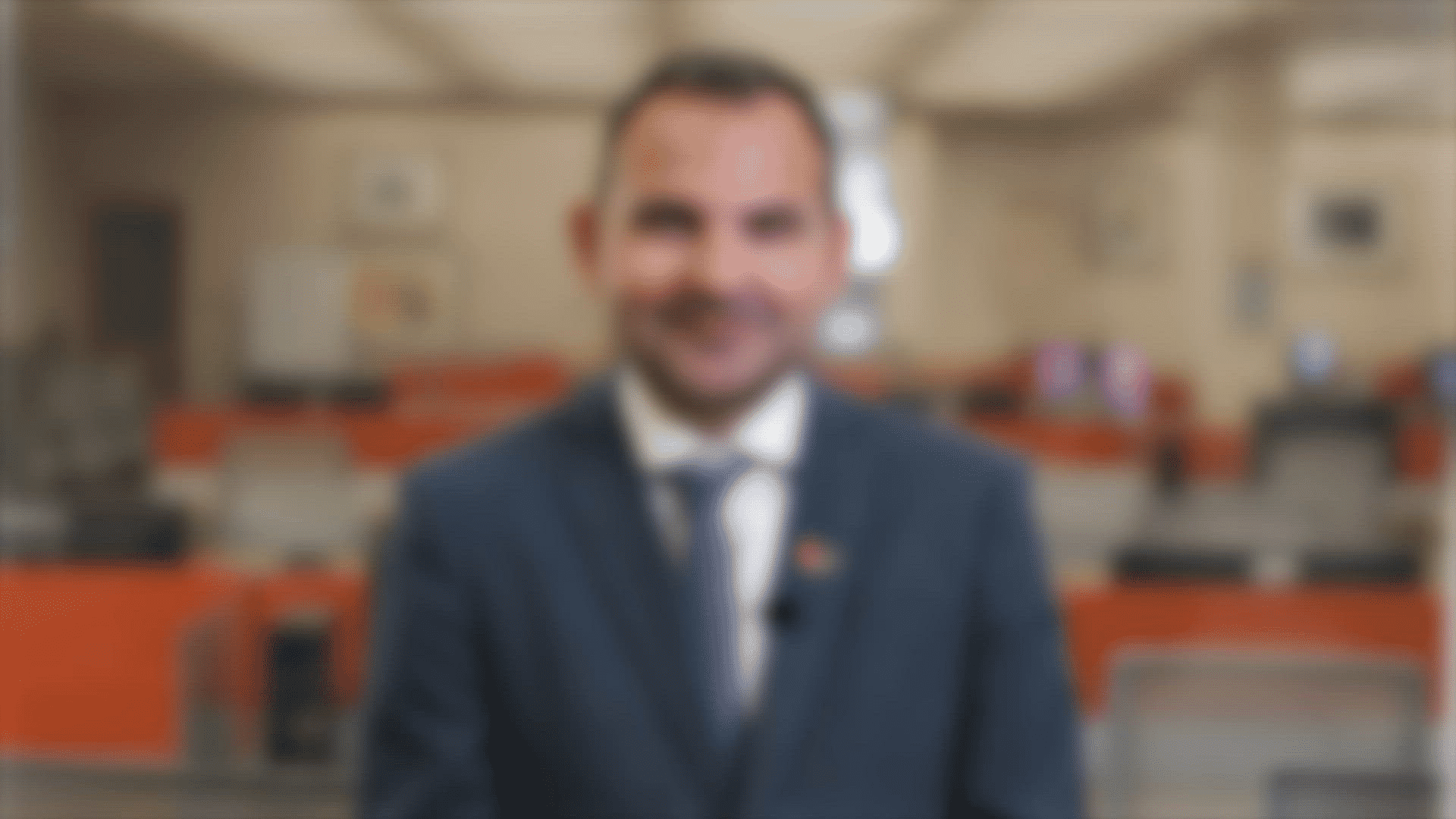

Supplement: Supplementary file 1 — AI in Medicine folderPre- and Posttest.docxFeedback Survey.docx [file mep_2374-8265.11524-s001.zip › A. AI in Medicine/assets/Dr. Chatzizisis.jpg]

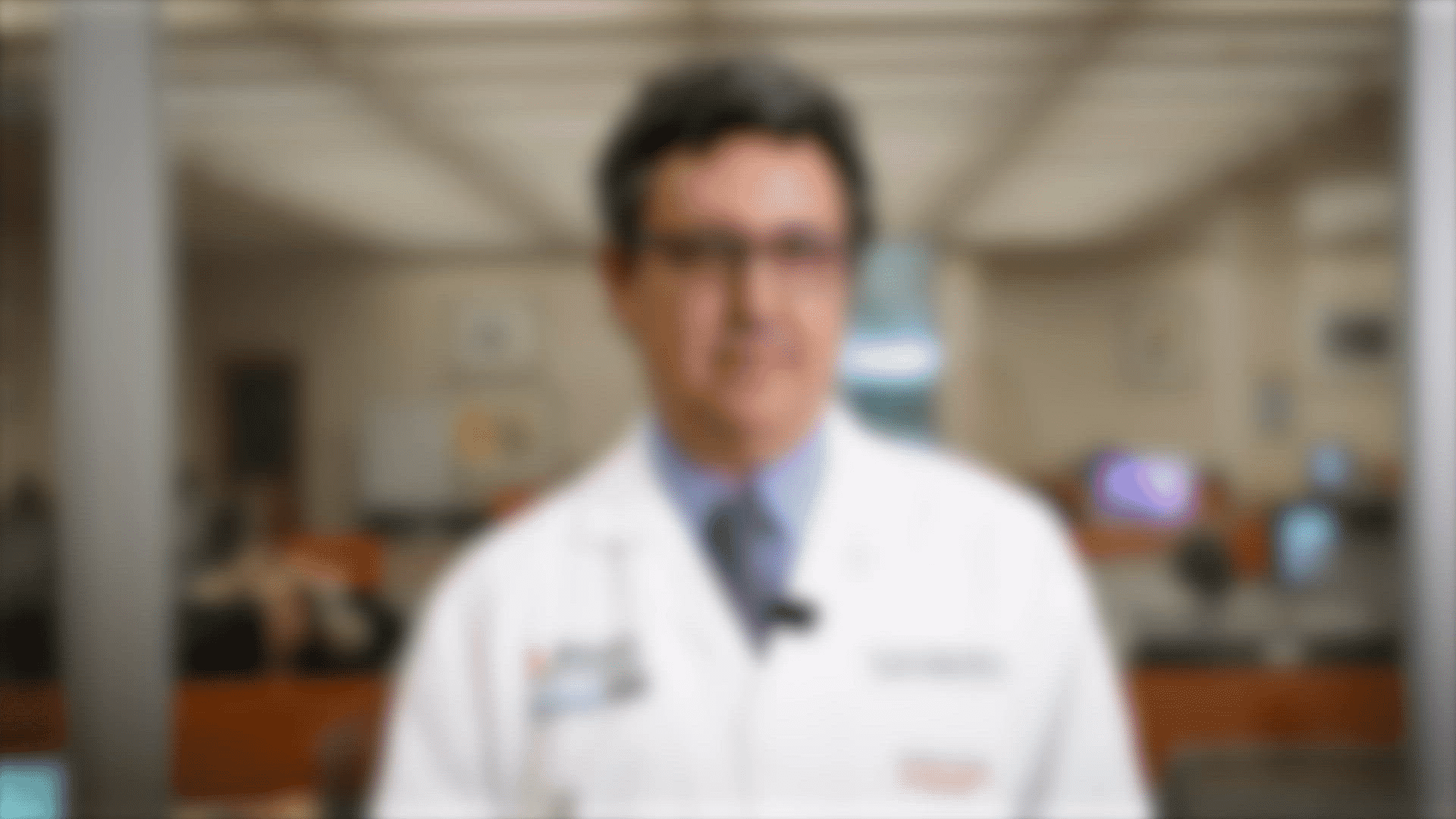

Supplement: Supplementary file 1 — AI in Medicine folderPre- and Posttest.docxFeedback Survey.docx [file mep_2374-8265.11524-s001.zip › A. AI in Medicine/assets/Dr. Collado-Mesa (1).jpg]

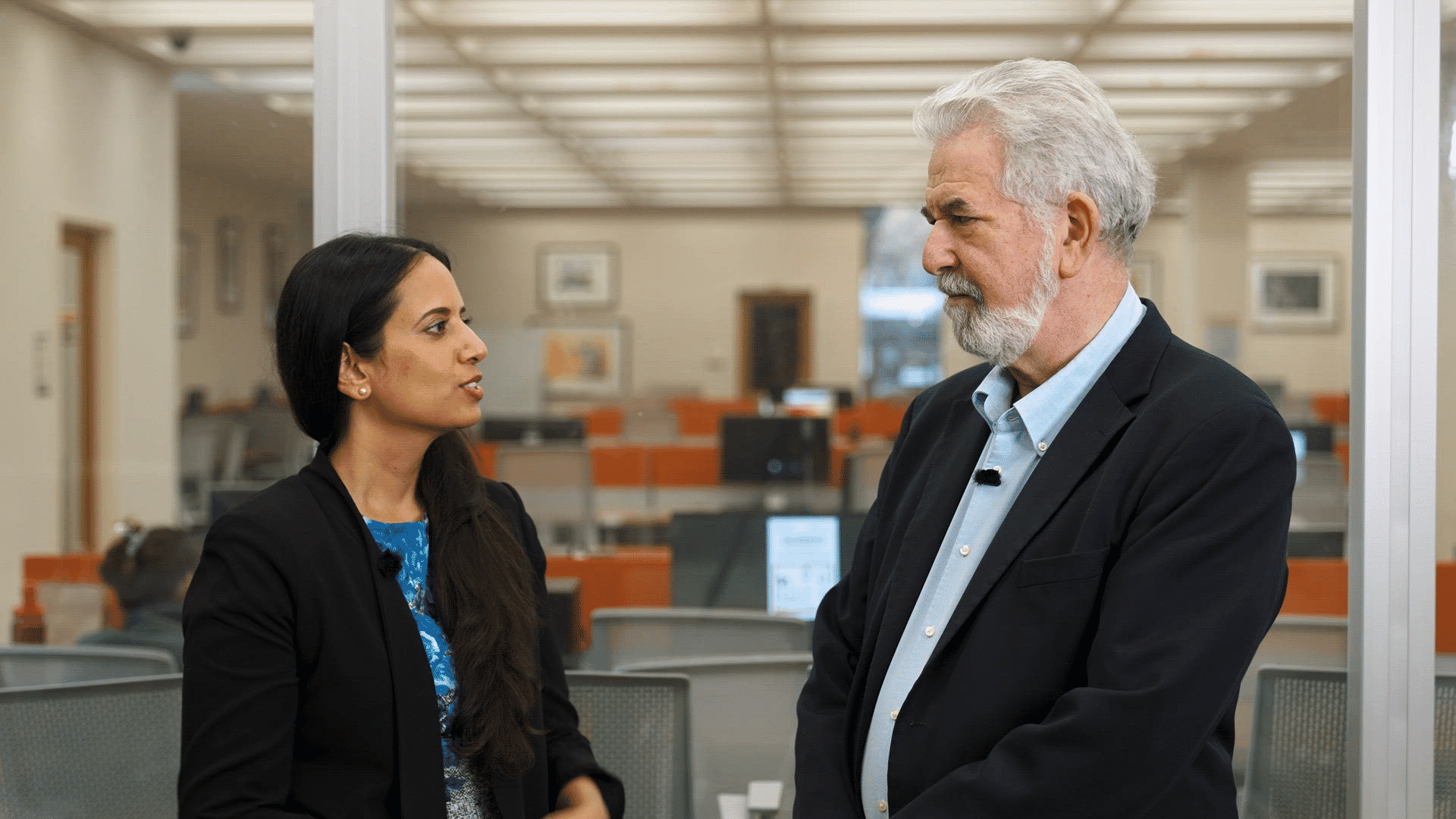

Supplement: Supplementary file 1 — AI in Medicine folderPre- and Posttest.docxFeedback Survey.docx [file mep_2374-8265.11524-s001.zip › A. AI in Medicine/assets/Dr. Goodman.jpg]

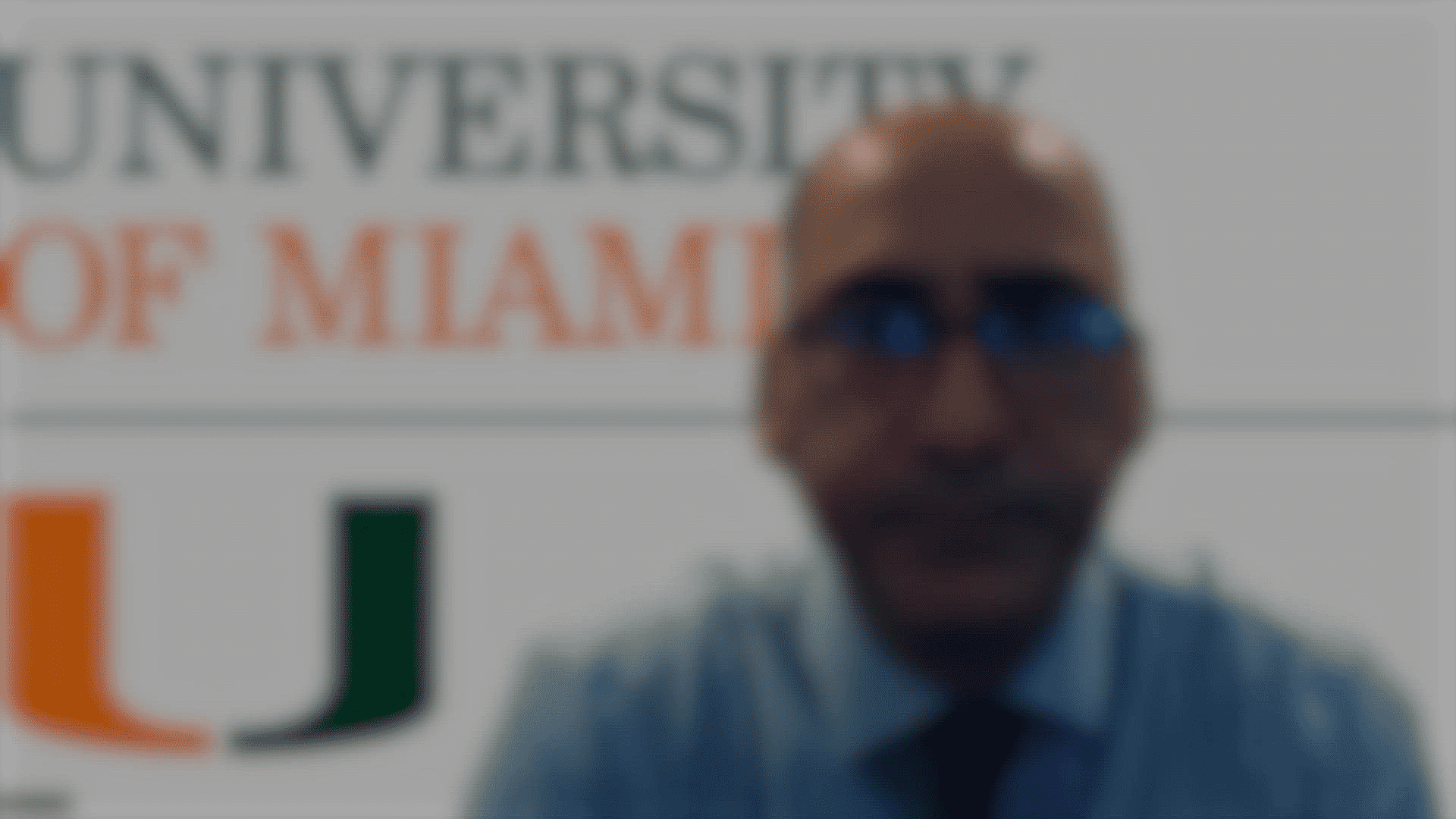

Supplement: Supplementary file 1 — AI in Medicine folderPre- and Posttest.docxFeedback Survey.docx [file mep_2374-8265.11524-s001.zip › A. AI in Medicine/assets/Dr. Jose.jpg]

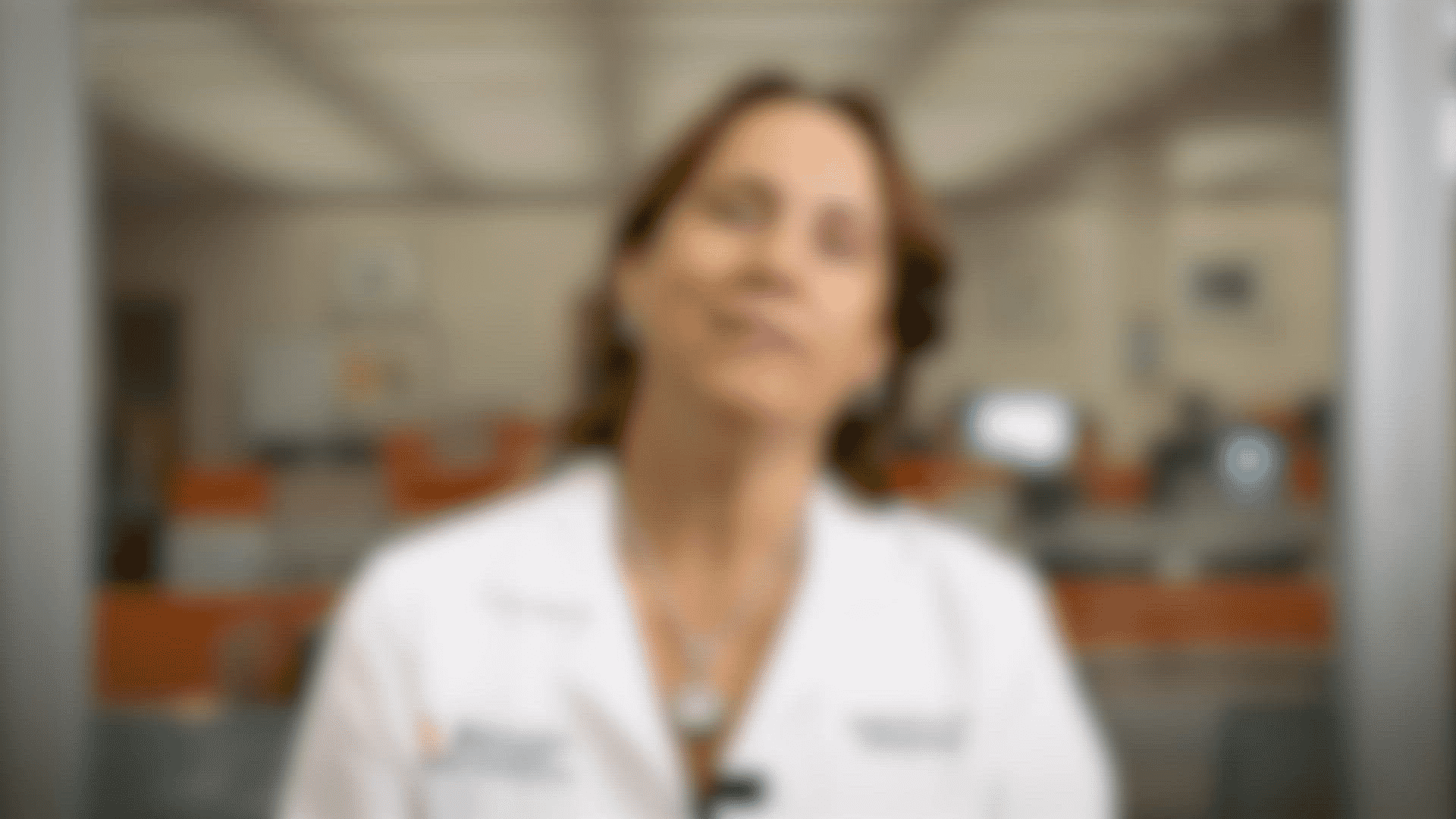

Supplement: Supplementary file 1 — AI in Medicine folderPre- and Posttest.docxFeedback Survey.docx [file mep_2374-8265.11524-s001.zip › A. AI in Medicine/assets/Dr. Ricur (1).jpg]

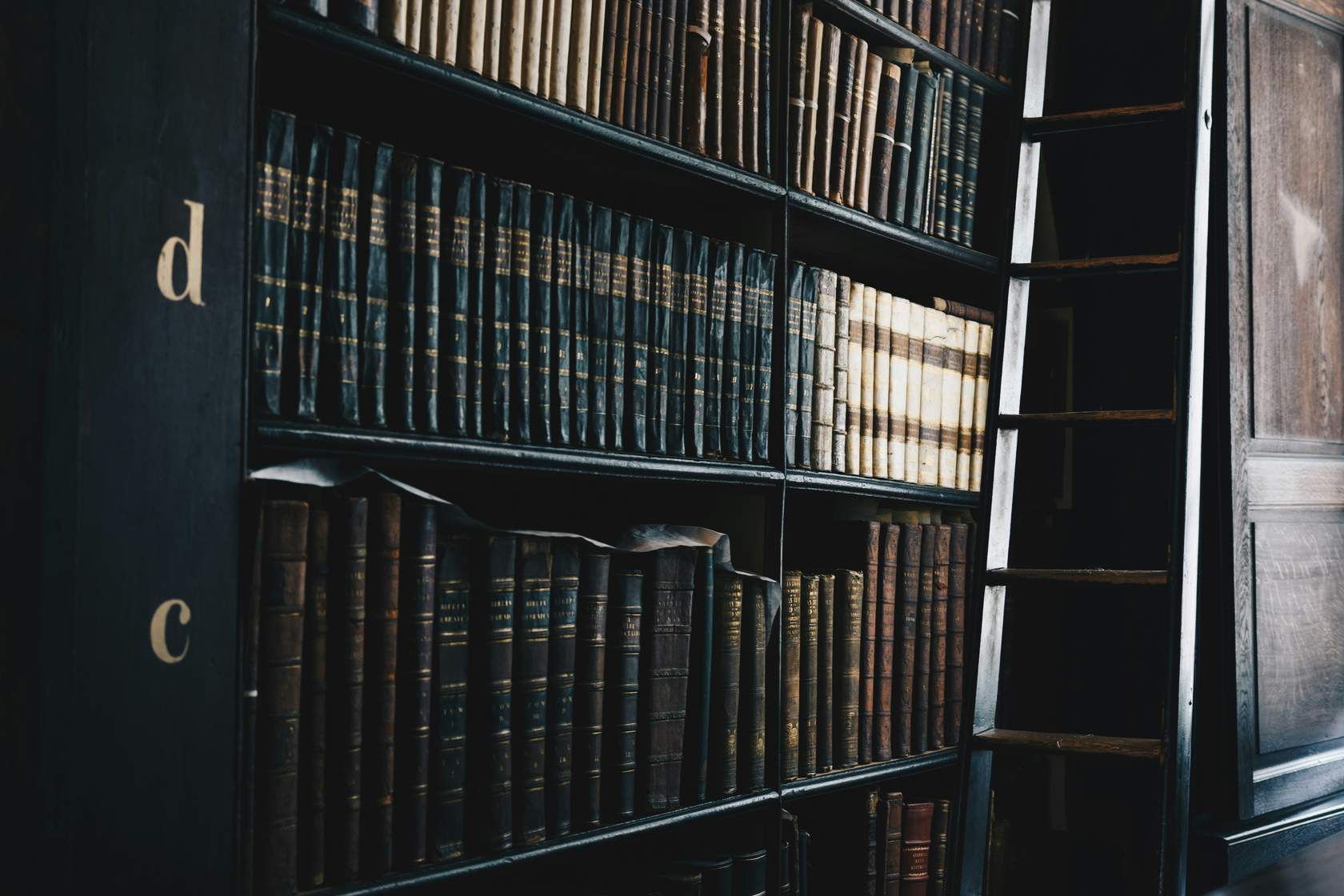

Supplement: Supplementary file 1 — AI in Medicine folderPre- and Posttest.docxFeedback Survey.docx [file mep_2374-8265.11524-s001.zip › A. AI in Medicine/assets/edQltt/stock-image.jpg]

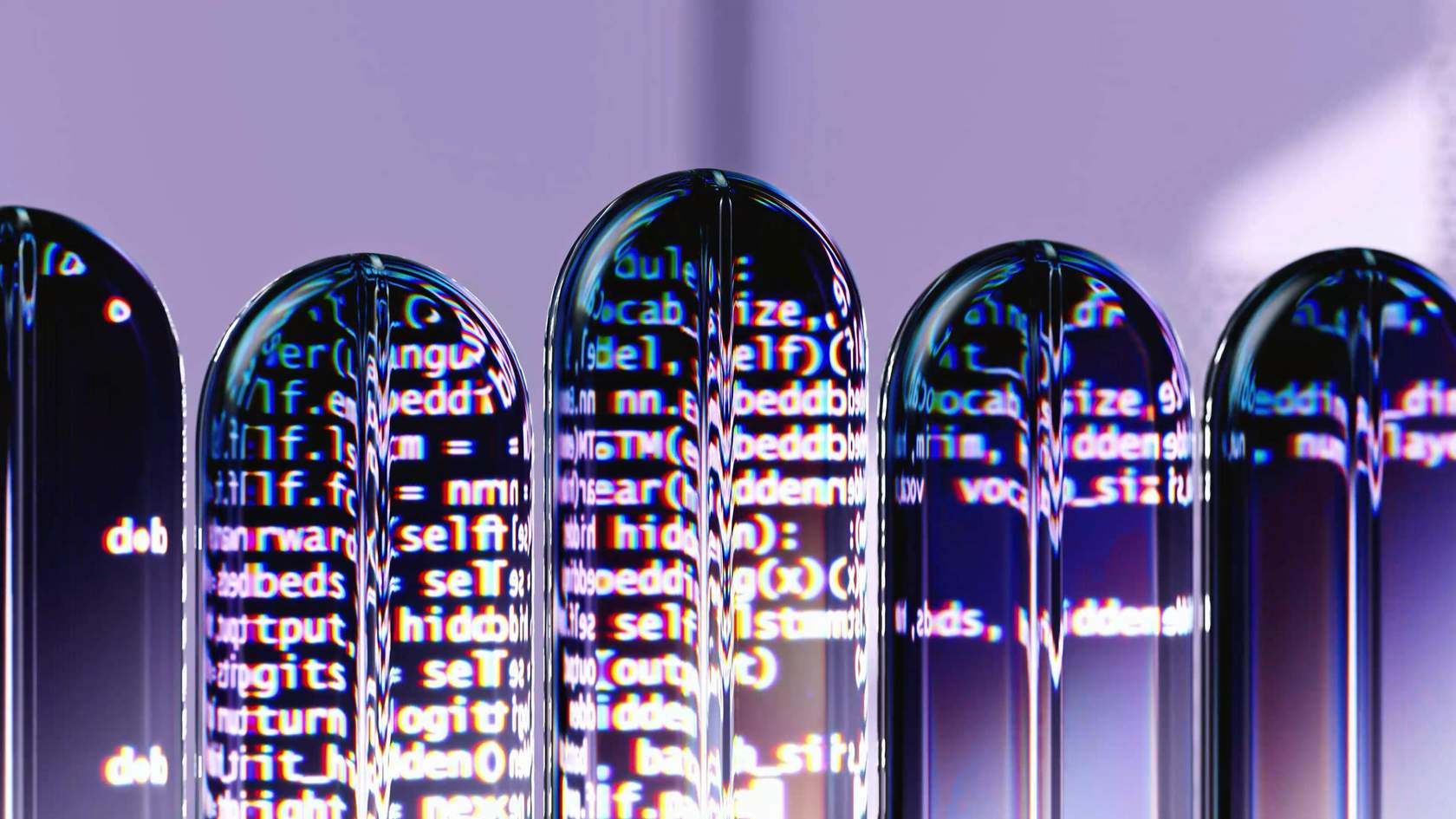

Supplement: Supplementary file 1 — AI in Medicine folderPre- and Posttest.docxFeedback Survey.docx [file mep_2374-8265.11524-s001.zip › A. AI in Medicine/assets/Fwx1k3/stock-image.jpg]

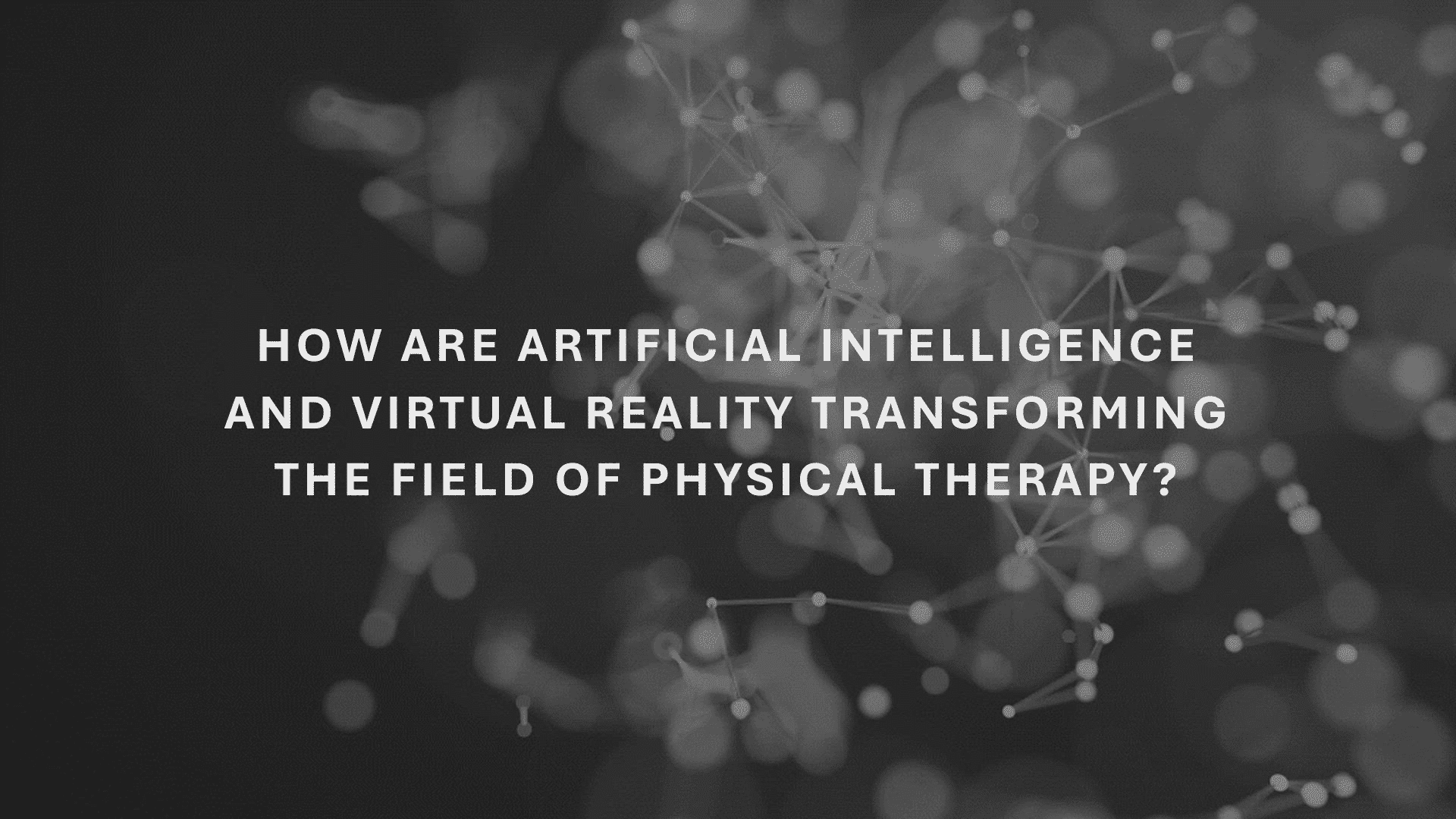

Supplement: Supplementary file 1 — AI in Medicine folderPre- and Posttest.docxFeedback Survey.docx [file mep_2374-8265.11524-s001.zip › A. AI in Medicine/assets/How are Artificial Intelligence and Virtual Reality.jpg]

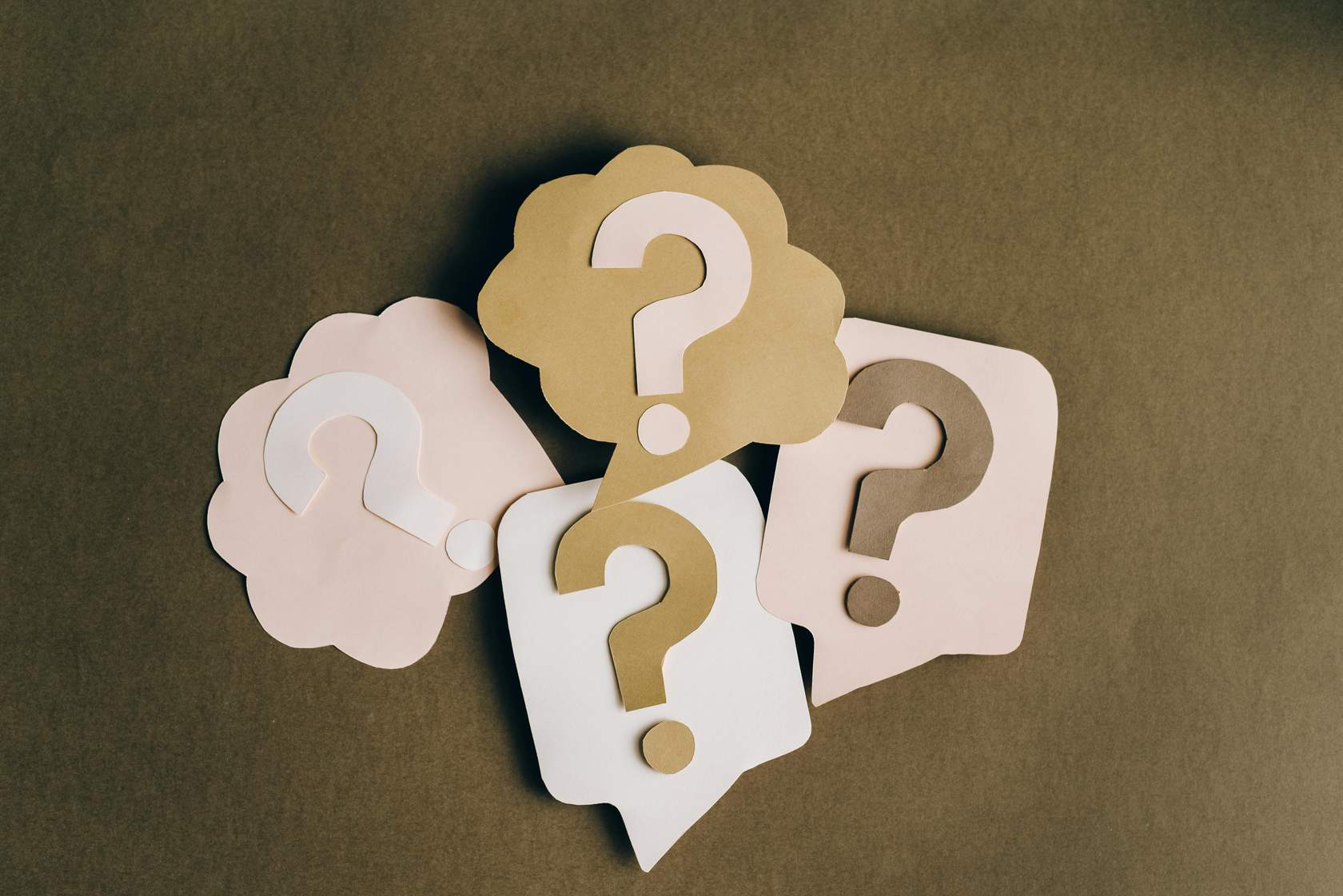

Supplement: Supplementary file 1 — AI in Medicine folderPre- and Posttest.docxFeedback Survey.docx [file mep_2374-8265.11524-s001.zip › A. AI in Medicine/assets/I-pnr0/stock-image.jpg]

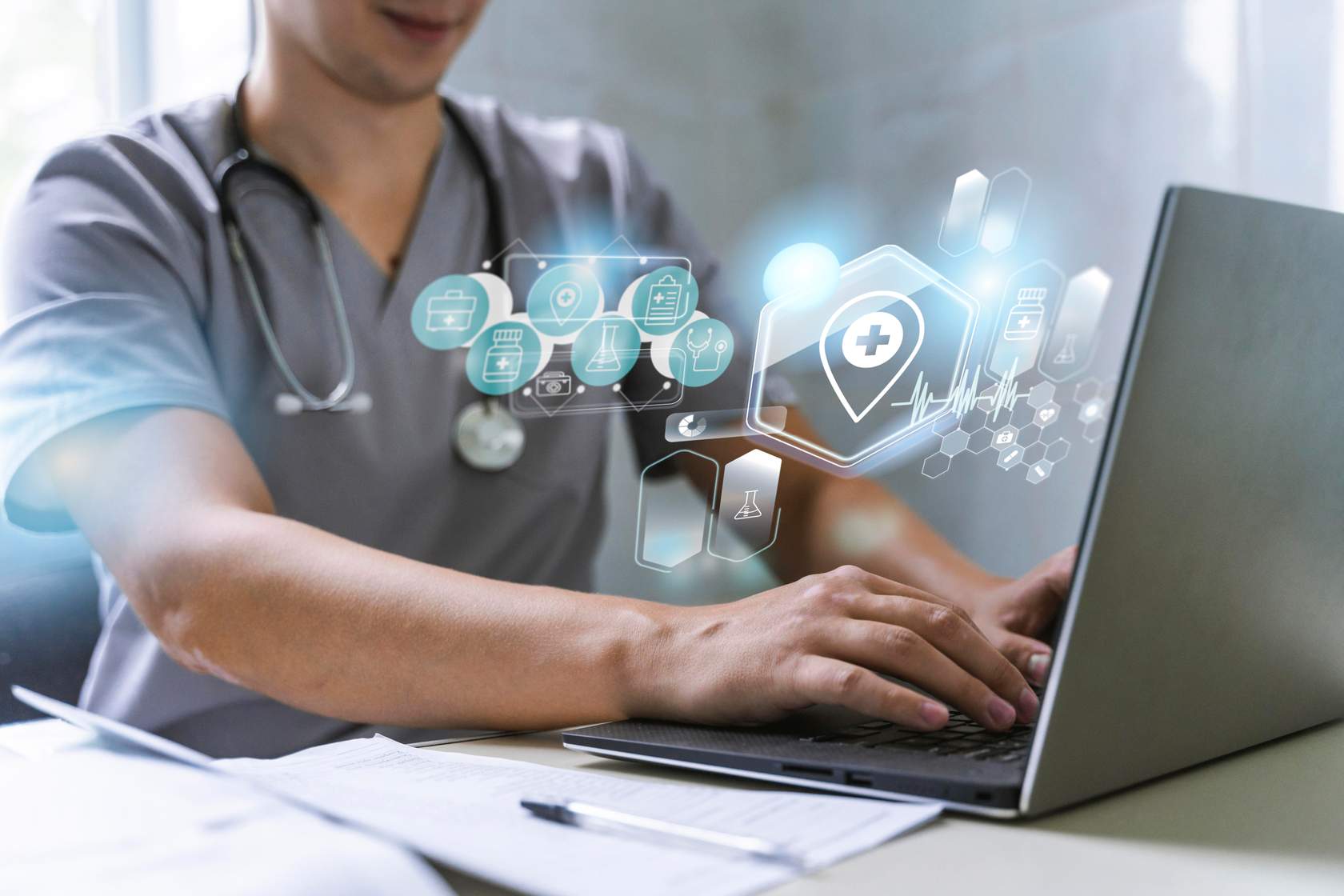

Supplement: Supplementary file 1 — AI in Medicine folderPre- and Posttest.docxFeedback Survey.docx [file mep_2374-8265.11524-s001.zip › A. AI in Medicine/assets/medical-banner-with-doctor-working-laptop.jpg]

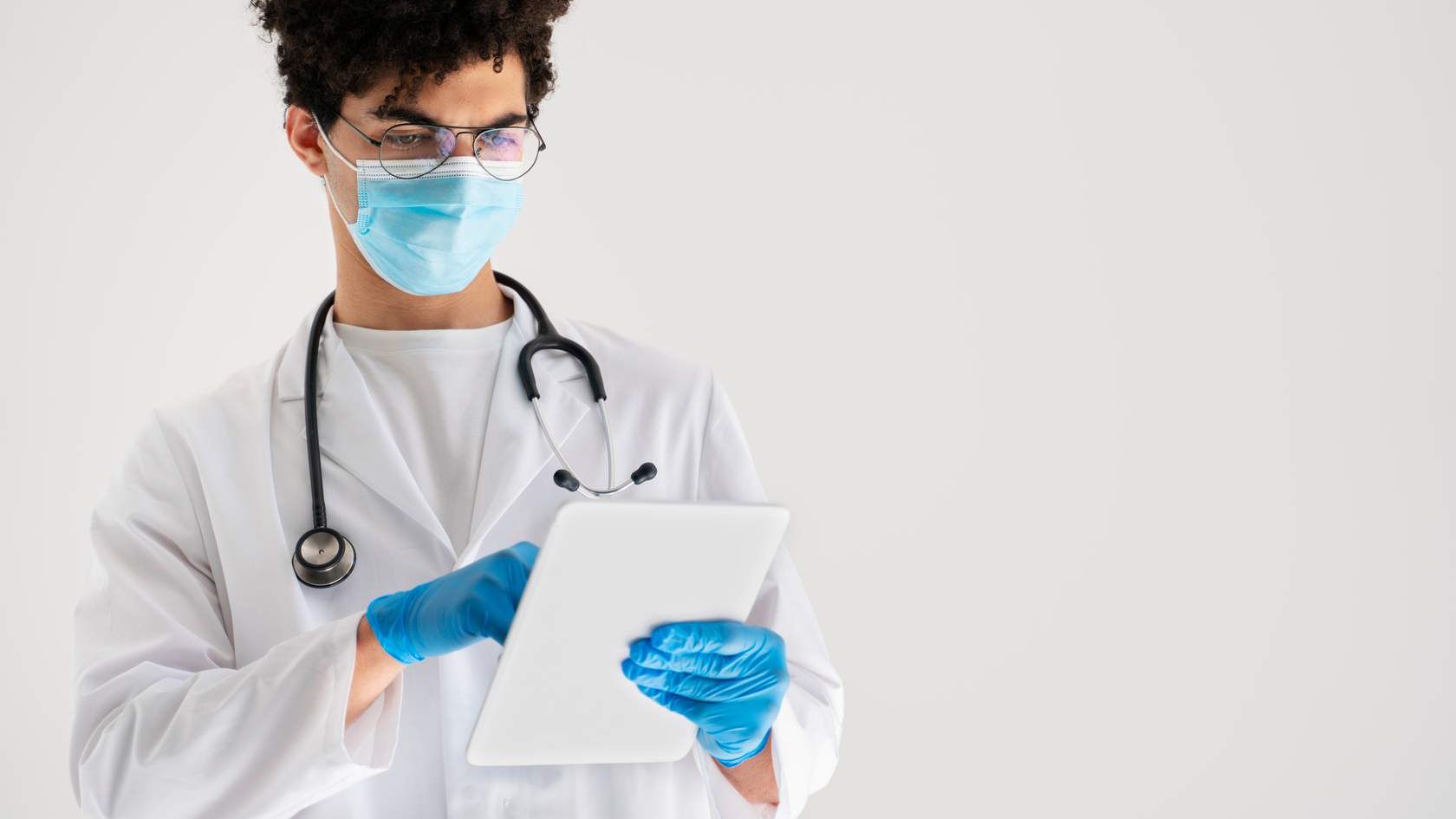

Supplement: Supplementary file 1 — AI in Medicine folderPre- and Posttest.docxFeedback Survey.docx [file mep_2374-8265.11524-s001.zip › A. AI in Medicine/assets/medium-shot-doctor-holding-tablet.jpg]

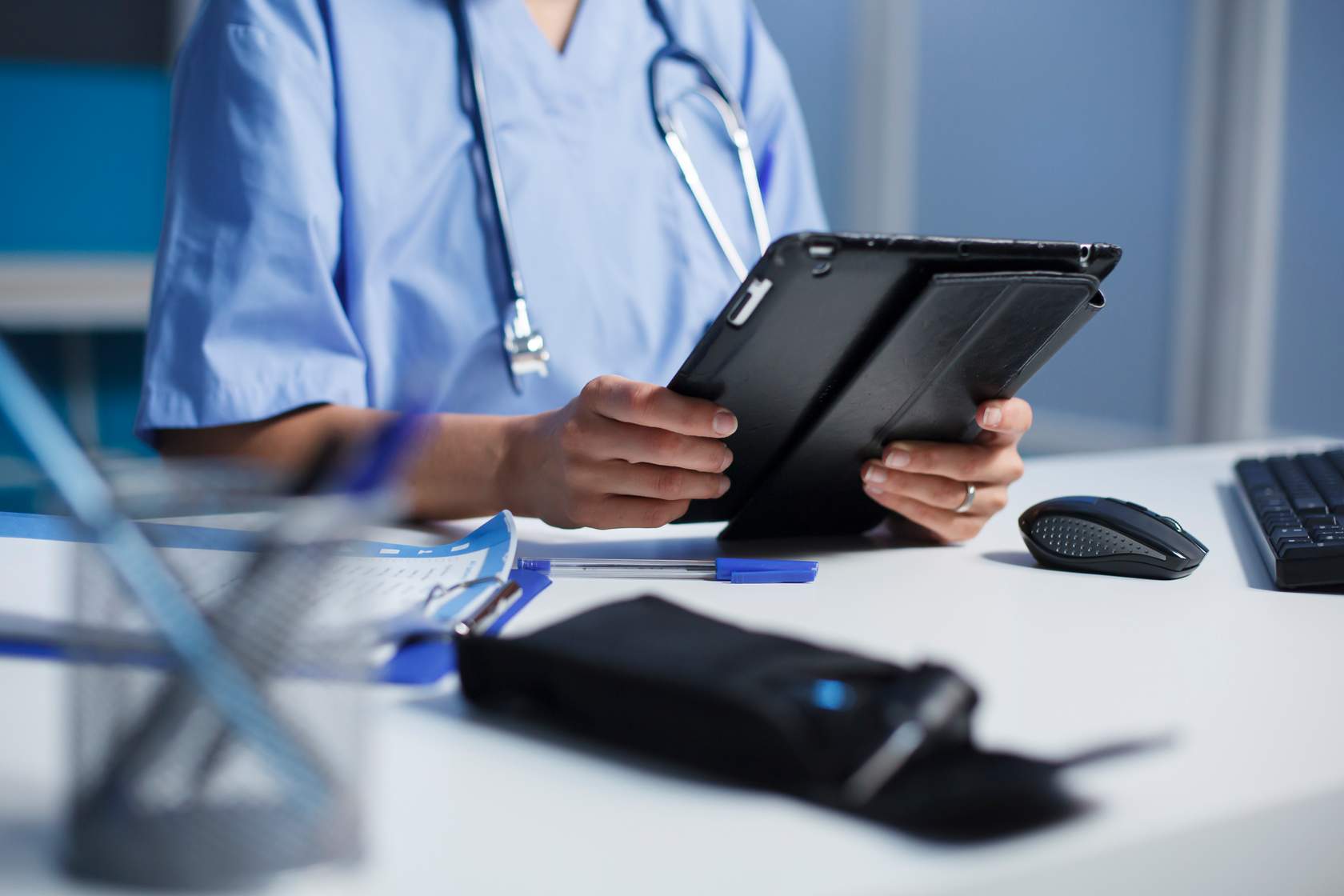

Supplement: Supplementary file 1 — AI in Medicine folderPre- and Posttest.docxFeedback Survey.docx [file mep_2374-8265.11524-s001.zip › A. AI in Medicine/assets/modern-hospital-office-using-devices.jpg]

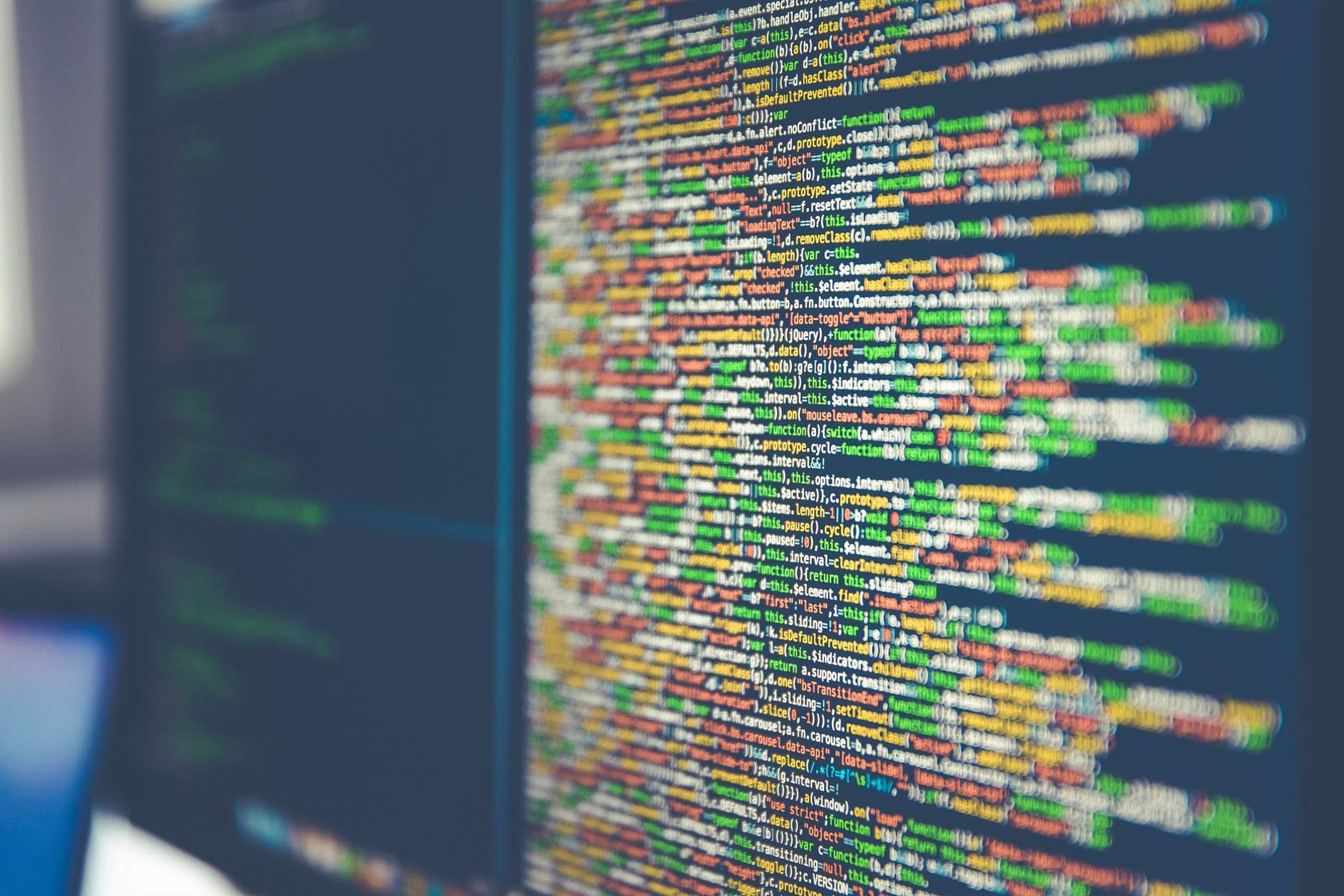

Supplement: Supplementary file 1 — AI in Medicine folderPre- and Posttest.docxFeedback Survey.docx [file mep_2374-8265.11524-s001.zip › A. AI in Medicine/assets/P7dZNuy_YE3cGYtL.jpg]

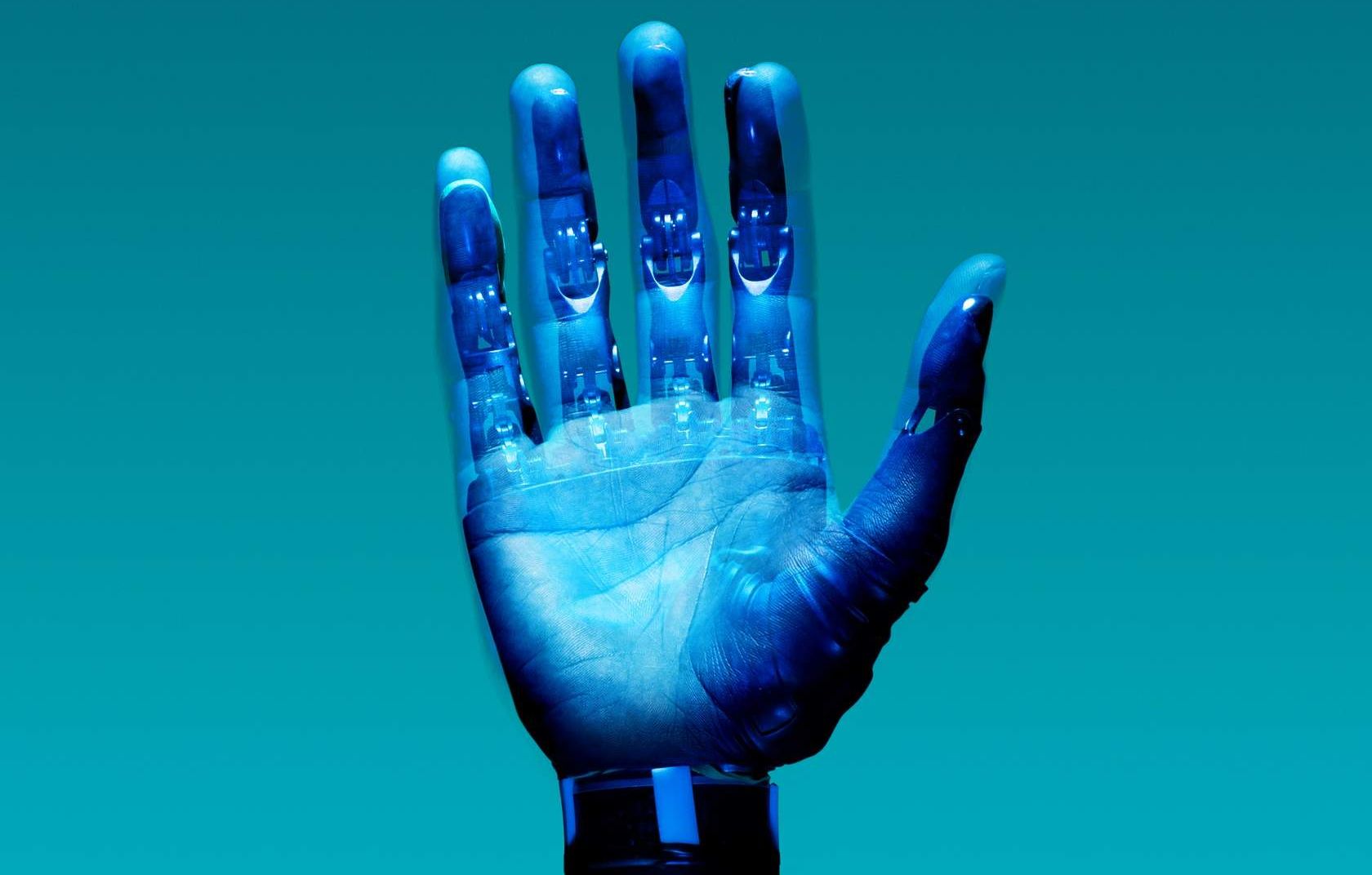

Supplement: Supplementary file 1 — AI in Medicine folderPre- and Posttest.docxFeedback Survey.docx [file mep_2374-8265.11524-s001.zip › A. AI in Medicine/assets/qcM8D5/stock-image.jpg]

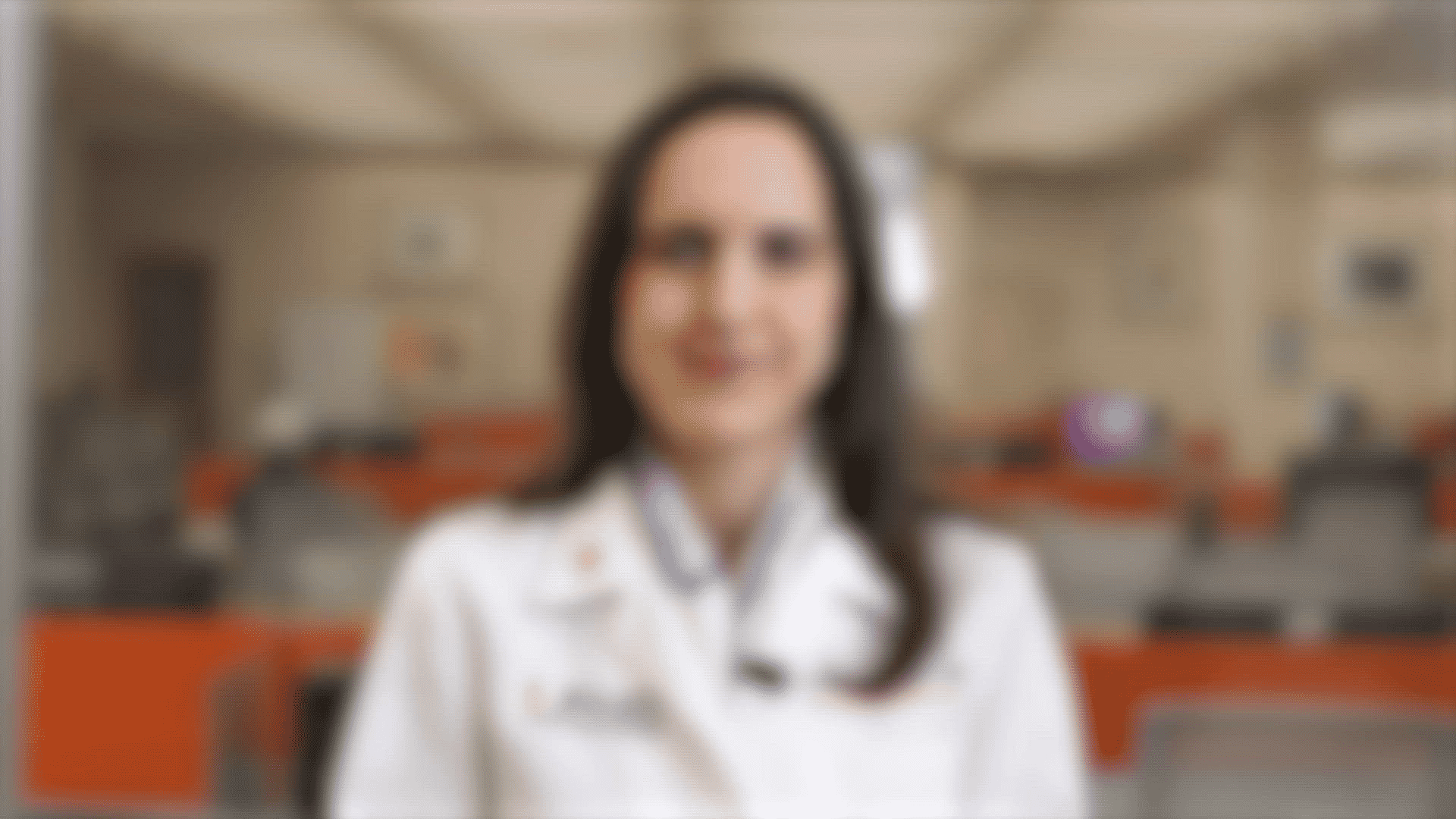

Supplement: Supplementary file 1 — AI in Medicine folderPre- and Posttest.docxFeedback Survey.docx [file mep_2374-8265.11524-s001.zip › A. AI in Medicine/assets/Sabrina.jpg]

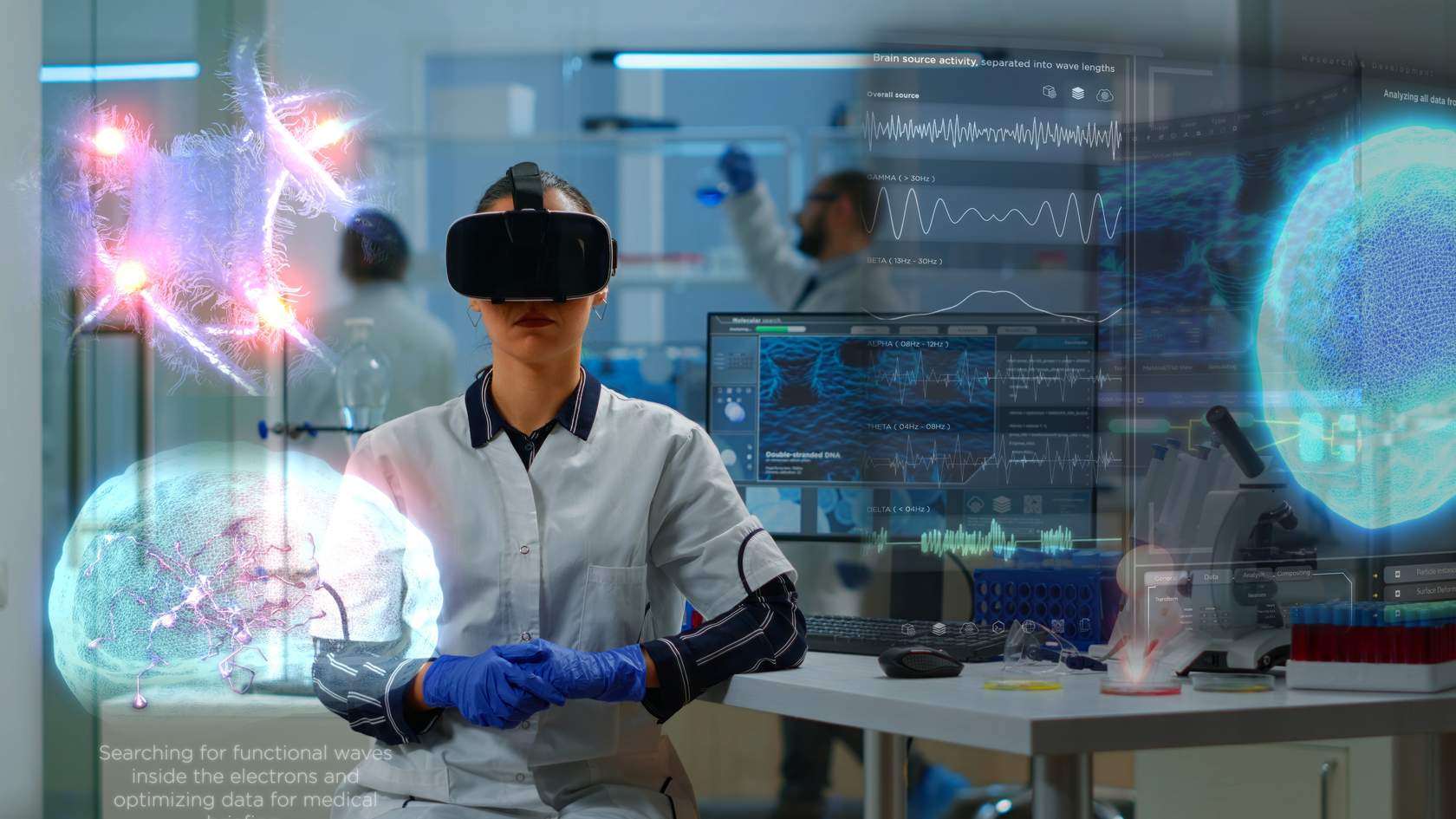

Supplement: Supplementary file 1 — AI in Medicine folderPre- and Posttest.docxFeedback Survey.docx [file mep_2374-8265.11524-s001.zip › A. AI in Medicine/assets/scientific-specialist-research-lab-wearing-vr.jpg]

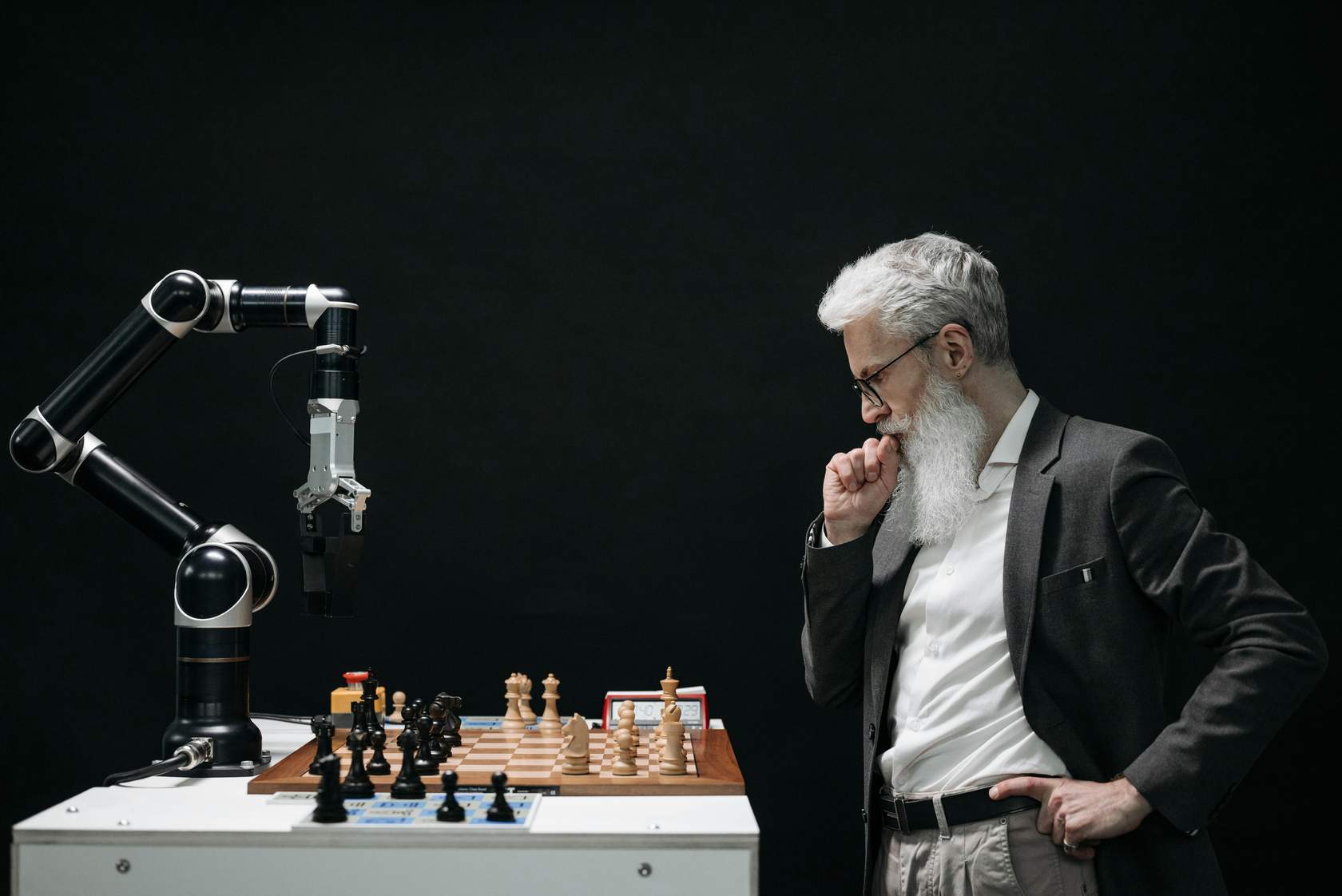

Supplement: Supplementary file 1 — AI in Medicine folderPre- and Posttest.docxFeedback Survey.docx [file mep_2374-8265.11524-s001.zip › A. AI in Medicine/assets/stock-image.jpg]

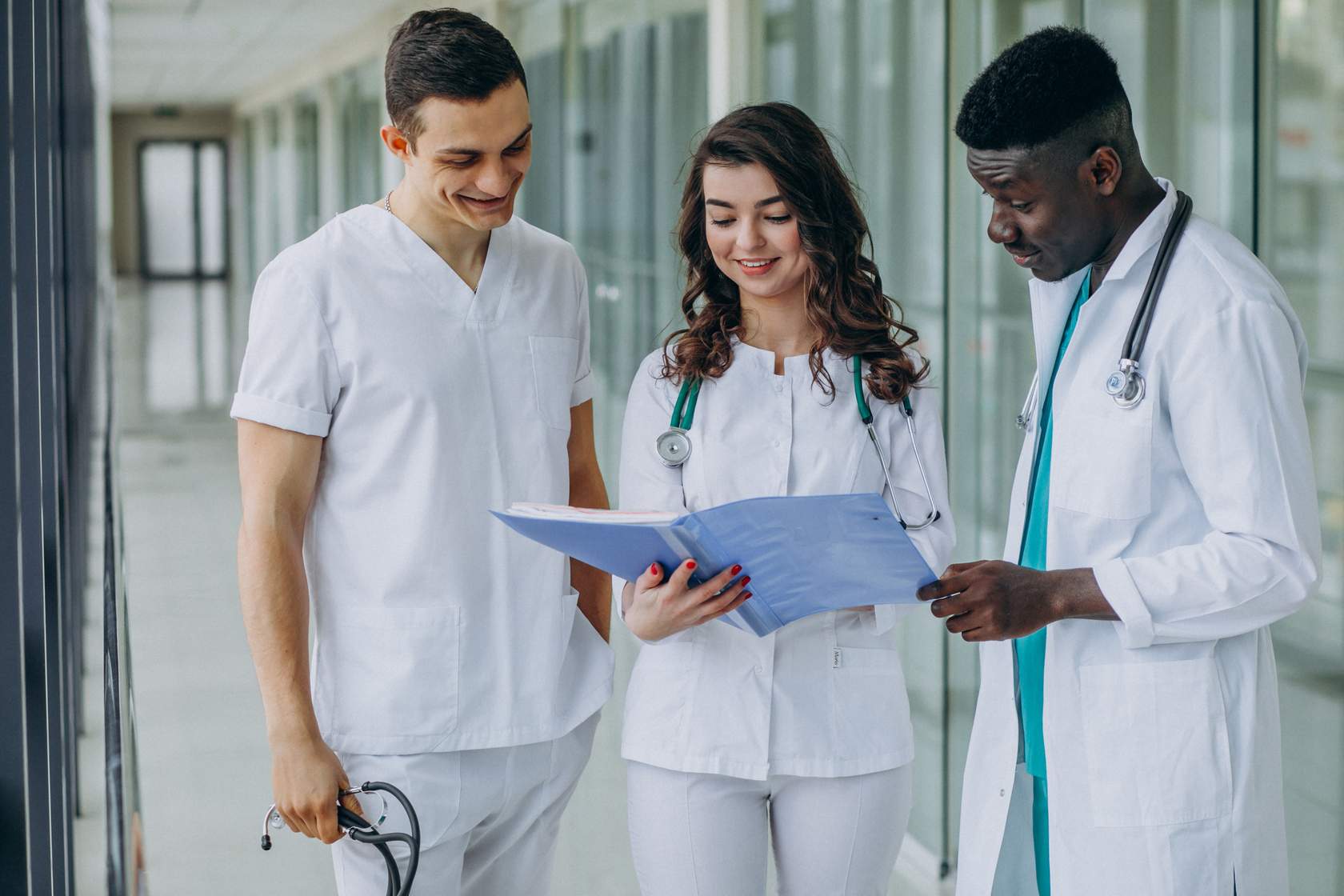

Supplement: Supplementary file 1 — AI in Medicine folderPre- and Posttest.docxFeedback Survey.docx [file mep_2374-8265.11524-s001.zip › A. AI in Medicine/assets/team-young-specialist-doctors-reviewing-documents-corridor.jpg]

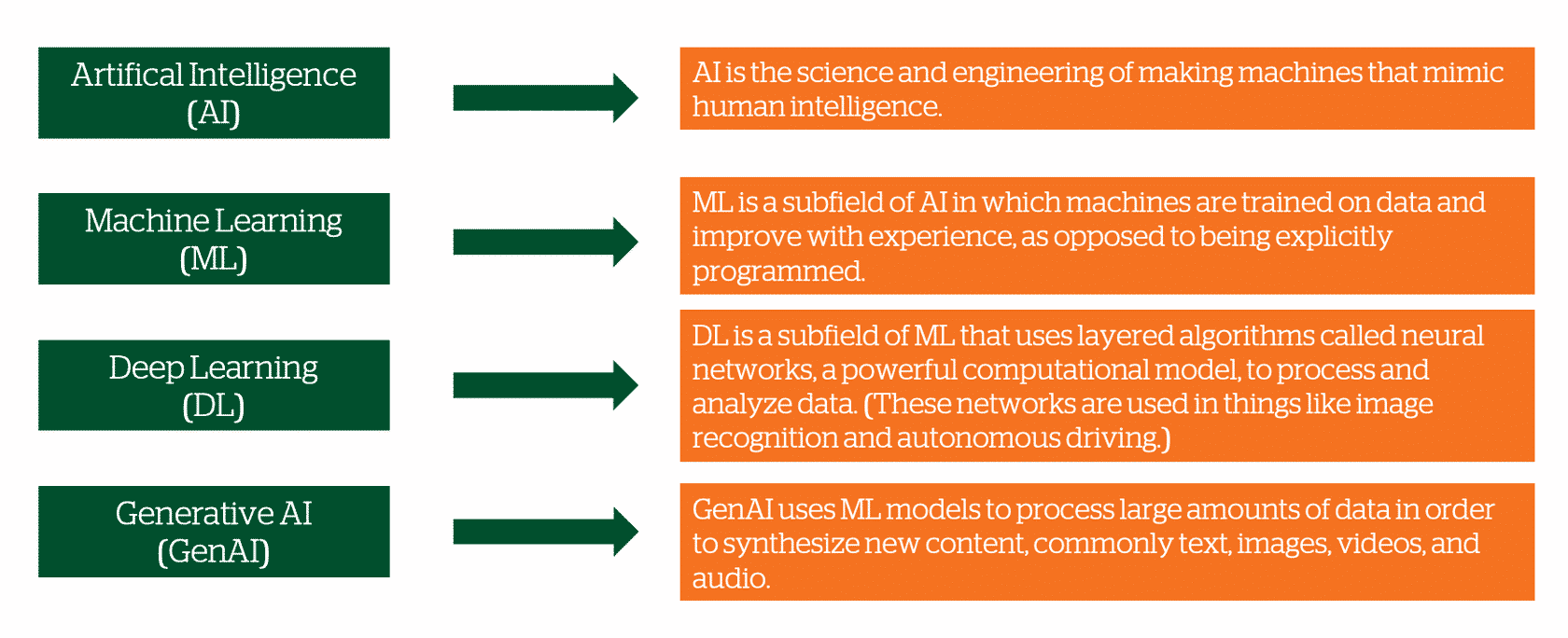

Supplement: Supplementary file 1 — AI in Medicine folderPre- and Posttest.docxFeedback Survey.docx [file mep_2374-8265.11524-s001.zip › A. AI in Medicine/assets/TERMS.png]

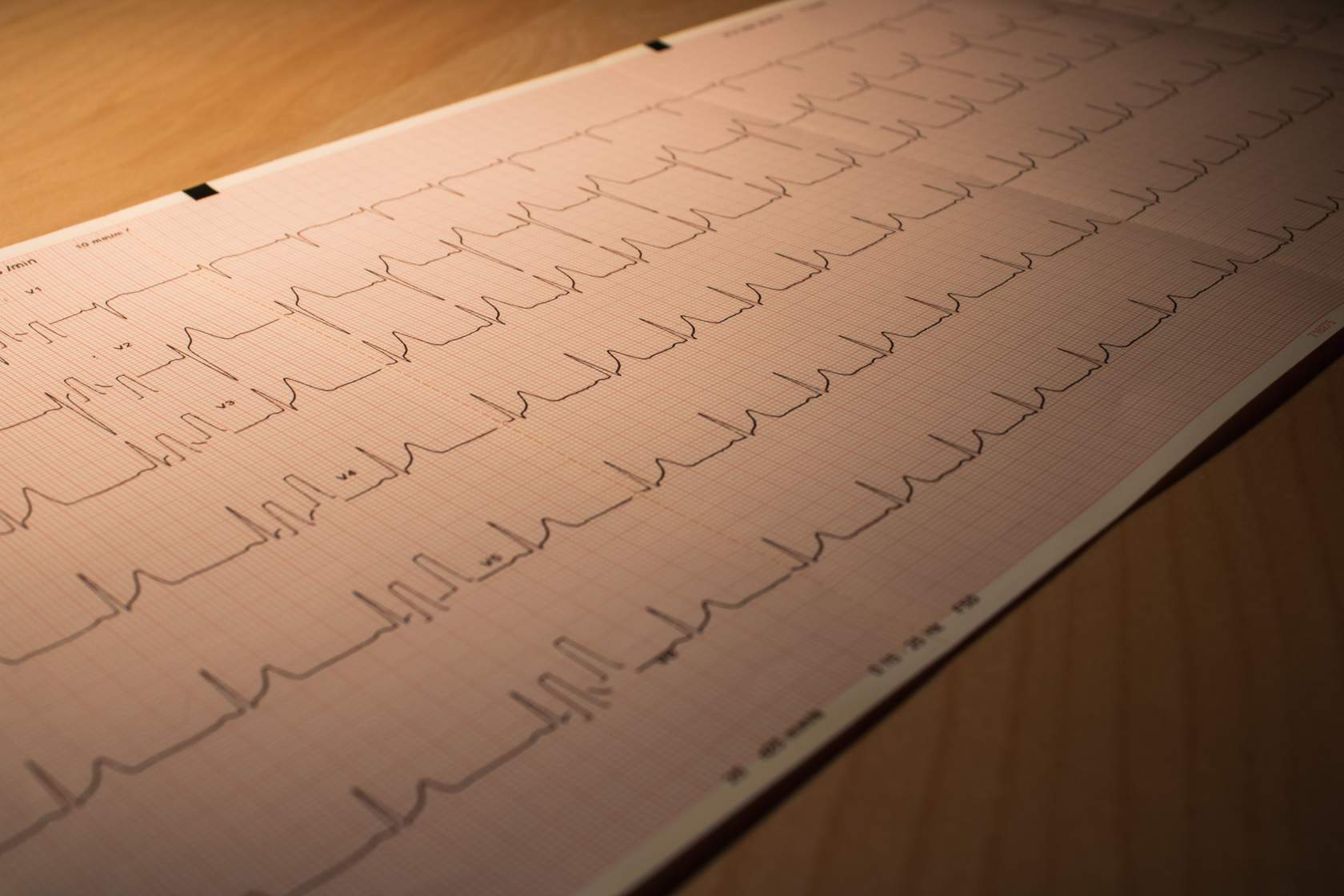

Supplement: Supplementary file 1 — AI in Medicine folderPre- and Posttest.docxFeedback Survey.docx [file mep_2374-8265.11524-s001.zip › A. AI in Medicine/assets/TryLd6/stock-image.jpg]

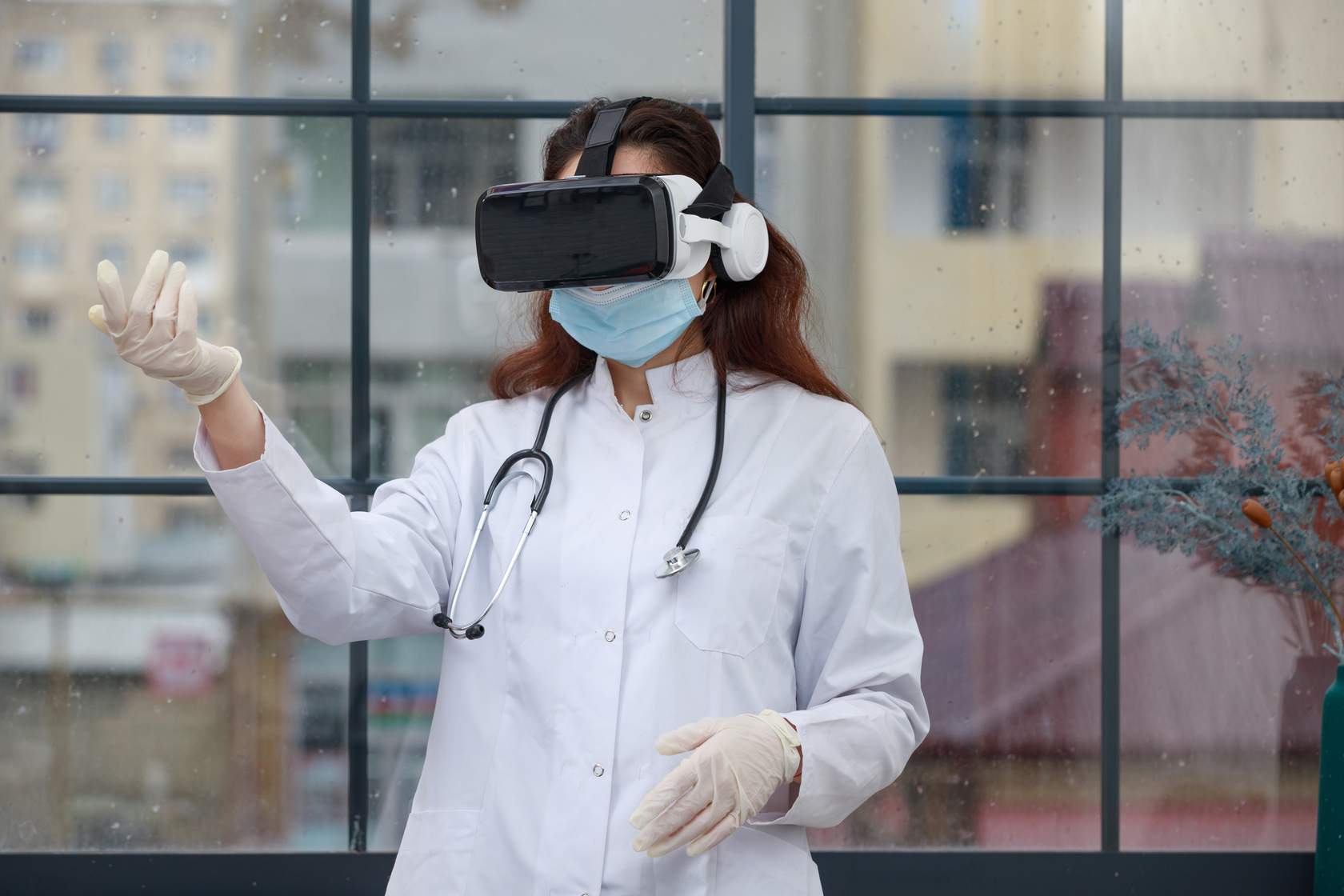

Supplement: Supplementary file 1 — AI in Medicine folderPre- and Posttest.docxFeedback Survey.docx [file mep_2374-8265.11524-s001.zip › A. AI in Medicine/assets/young-female-doctor-wearing-vr-glasses-holding.jpg]

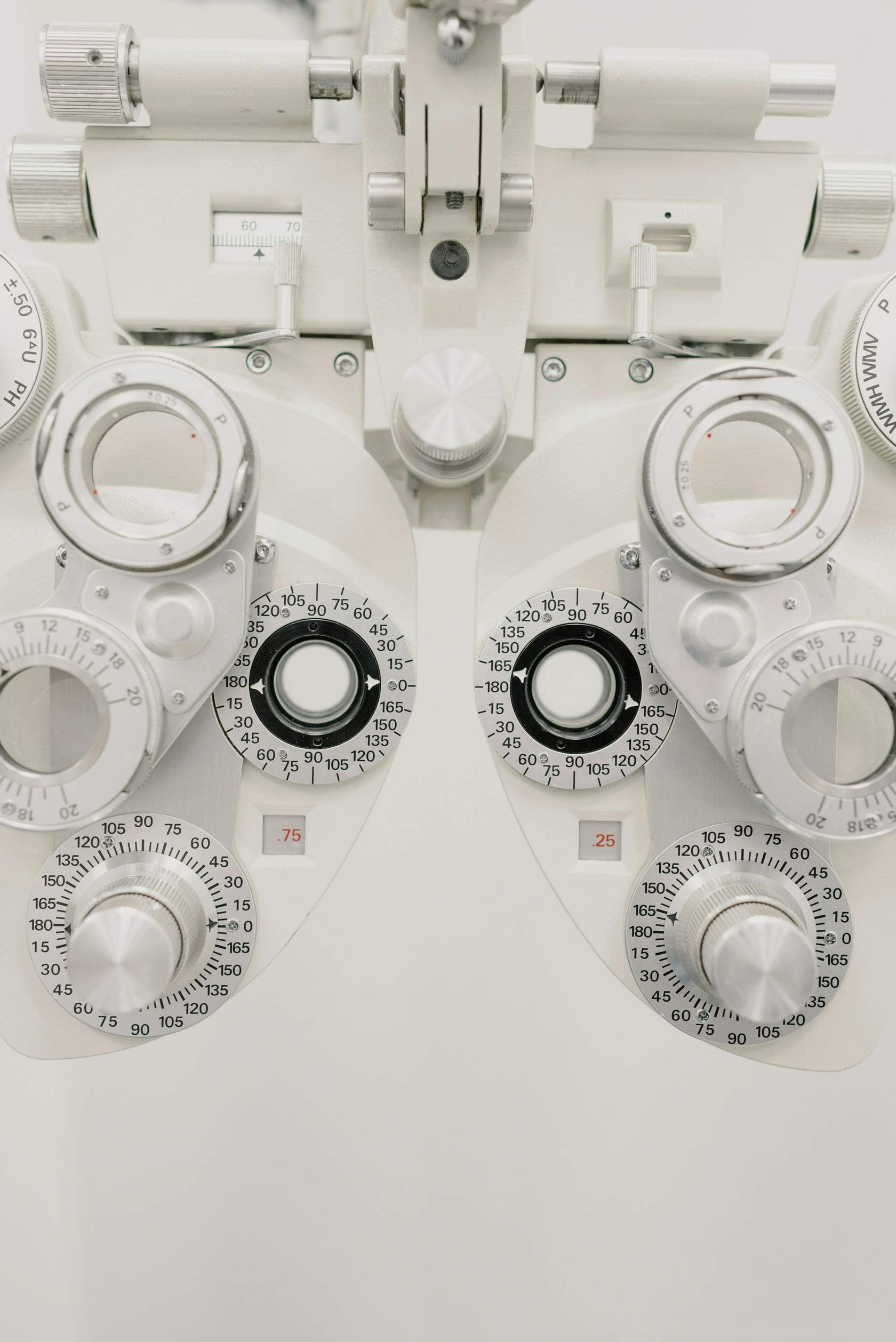

Supplement: Supplementary file 1 — AI in Medicine folderPre- and Posttest.docxFeedback Survey.docx [file mep_2374-8265.11524-s001.zip › A. AI in Medicine/assets/_7DChH/stock-image.jpg]
